# Multi-locus genome-wide association studies reveal the genetic architecture of *Fusarium* head blight resistance in durum wheat

**DOI:** 10.3389/fpls.2023.1182548

**Published:** 2023-10-12

**Authors:** Jemanesh K. Haile, Demissew Sertse, Amidou N’Diaye, Valentyna Klymiuk, Krystalee Wiebe, Yuefeng Ruan, Harmeet S. Chawla, Maria-Antonia Henriquez, Lipu Wang, Hadley R. Kutcher, Barbara Steiner, Hermann Buerstmayr, Curtis J. Pozniak

**Affiliations:** ^1^ Department of Plant Sciences, Crop Development Centre, University of Saskatchewan, Saskatoon, SK, Canada; ^2^ Aquatic and Crop Resource Development, National Research Council Canada, Saskatoon, SK, Canada; ^3^ Swift Current Research and Development Centre, Agriculture and Agri-Food Canada, Swift Current, SK, Canada; ^4^ Department of Plant Sciences, University of Manitoba, Winnipeg, MB, Canada; ^5^ Morden Research and Development Centre, Agriculture and Agri-Food Canada, Morden, MB, Canada; ^6^ Department of Agrobiotechnology, Institute of Biotechnology in Plant Production, University of Natural Resources and Life Sciences Vienna, Tulln, Austria

**Keywords:** durum wheat, FHB resistance, DON, GWAS, multi-locus, GDP, KASP markers

## Abstract

Durum wheat is more susceptible to Fusarium head blight (FHB) than other types or classes of wheat. The disease is one of the most devastating in wheat; it reduces yield and end-use quality and contaminates the grain with fungal mycotoxins such as deoxynivalenol (DON). A panel of 265 Canadian and European durum wheat cultivars, as well as breeding and experimental lines, were tested in artificially inoculated field environments (2019–2022, inclusive) and two greenhouse trials (2019 and 2020). The trials were assessed for FHB severity and incidence, visual rating index, *Fusarium*-damaged kernels, DON accumulation, anthesis or heading date, maturity date, and plant height. In addition, yellow pigment and protein content were analyzed for the 2020 field season. To capture loci underlying FHB resistance and related traits, GWAS was performed using single-locus and several multi-locus models, employing 13,504 SNPs. Thirty-one QTL significantly associated with one or more FHB-related traits were identified, of which nine were consistent across environments and associated with multiple FHB-related traits. Although many of the QTL were identified in regions previously reported to affect FHB, the QTL *QFhb-3B.2*, associated with FHB severity, incidence, and DON accumulation, appears to be novel. We developed KASP markers for six FHB-associated QTL that were consistently detected across multiple environments and validated them on the Global Durum Panel (GDP). Analysis of allelic diversity and the frequencies of these revealed that the lines in the GDP harbor between zero and six resistance alleles. This study provides a comprehensive assessment of the genetic basis of FHB resistance and DON accumulation in durum wheat. Accessions with multiple favorable alleles were identified and will be useful genetic resources to improve FHB resistance in durum breeding programs through marker-assisted recurrent selection and gene stacking.

## 1 Introduction

According to FAO, food production needs to increase by 70% (baseline 2009) to feed a growing world population, which is projected to reach ~9.1 billion by 2050 (FAO, 2009). Reports that considered recent consumption behaviors and updated 2050 population projections (~10 billion) estimate that crop production of food crops will need to be increased by 119% to meet the demand (Berners-Lee et al., 2018). While achieving this production goal is feasible, major biotic and abiotic constraints further constrain crop production. For example, diseases and other pests account for up to 40% of annual yield loss in crop production (Savary et al., 2012). The prevalence of diseases and other pests has been exacerbated by increasing climate-change-related burdens and world trade and movements (Carvajal-Yepes et al., 2019; Prank et al., 2019).

Wheat is the most important cereal crop in the world; it is produced on 217 million hectares globally and is a major source of nutrition and caloric intake (OECD/FAO, 2019). However, diseases and other pests heavily constrain wheat production in general and durum wheat in particular. An average yield loss in wheat due to biotic stresses is estimated to be over 20% a year (Savary et al., 2019). Most wheat diseases are fungal-caused, and genetic resistance has been effective in controlling several diseases, like wheat rust. However, *Fusarium* head blight (FHB) (Miedaner et al., 2017) remains one of the most destructive diseases of wheat worldwide, especially for durum wheat, because it is generally the most susceptible of the small-grained cereals. Breeding for FHB resistance is a priority, but it is hindered by its complex genetic architecture, significant genotype-by-environment interaction, and high cost of phenotype testing. In addition to direct yield losses caused by FHB, indirect losses due to the contamination of infected kernels with *Fusarium* mycotoxins are becoming a primary concern.

Mycotoxins are toxic substances that cause a significant annual economic loss to the agriculture and food industries. Each year, approximately 25% of agricultural commodities are contaminated by mycotoxins. One of the major mycotoxins in the wheat supply chain with a critical health risk potential is deoxynivalenol (DON). DON is a trichothecene mycotoxin produced by *Fusarium graminearum* and *Fusarium culmorum* (Mirocha et al., 1994). It is also known as vomitoxin, which is harmful to humans and animals after consumption (Foroud et al., 2019) because it can cause vomiting, anorexia, growth retardation, immune suppression, inflammation and necrosis of various tissues, and diarrhea in animals (Pestka and Smolinski, 2005). The Codex Committee on Contaminants in Food develops and proposes international food safety standards and codes of practice and has set the maximum DON allowable at 2.0 mg/kg for cereal grains, including wheat (Canadian Grain Commission, 2019). Therefore, breeding for FHB-resistant cultivars, together with an integrated disease management strategy, is the most effective, economical, and environment-friendly way to combat the disease globally.

Most current durum wheat cultivars are highly susceptible to FHB, and breeding progress is hampered by the narrow genetic variation for FHB resistance in elite germplasm. Compared to hexaploid wheat, fewer resistance loci are reported for FHB resistance in tetraploid wheat. Furthermore, most of these quantitative trait loci (QTL) possess only minor or moderate effects compared to the major resistance loci in hexaploid wheat, e.g., *Fhb1* located on chromosome 3BS, *Fhb2* located on chromosome 6BS, and *Qfhs.ifa-5A* are all derived from the resistant Chinese cultivar “Sumai 3” (Prat et al., 2014). Introgression of resistance genes from hexaploid into durum wheat has been largely unsuccessful, except for a recent report where introgression of *Fhb1* from Sumai 3 into durum wheat resulted in an improved resistance response (Giancaspro et al., 2016; Prat et al., 2017). In addition, FHB resistance in wheat is usually negatively associated with agronomic traits such as semi-dwarfness and other plant phenological traits, a fact that complicates the genetic mapping of resistance loci or deploying them in breeding. There is compelling evidence supporting a negative correlation between FHB resistance and plant height (PH) and heading date (HD), which is often reflected in the colocalization of PH and HD QTL with FHB resistance QTL (Buerstmayr et al., 2003; Buerstmayr et al., 2012; Sari et al., 2018; Sari et al., 2020). In addition, the widely deployed dwarfing allele *Rht-B1* has been associated with FHB susceptibility (Buerstmayr and Buerstmayr, 2016; He et al., 2016a). Buerstmayr et al. (2020) summarized the influence of PH, anther extrusion/retention, and HD/flowering time on FHB response. This has motivated the phenotyping of agro-morphological traits along with FHB resistance for most recent FHB studies (Haile et al., 2020).

Despite the lack of genetic diversity for FHB resistance, several studies have identified FHB resistance QTL derived from tetraploid wheat, suggesting the presence of minor-effect resistance that could be the focus of gene pyramiding strategies. Several genome-wide association studies (GWAS) have been reported using tetraploid wheat germplasm, including a Canadian durum wheat breeding panel (Sari et al., 2020), Tunisian-derived durum wheat populations (Ghavami et al., 2011), diverse durum lines from Northern America, the Mediterranean, Central Europe, Australia, and CIMMYT (Steiner et al., 2019b), and a durum association mapping panel mainly comprising Canadian breeding lines (Ruan et al., 2020). Most of the QTL mapping studies assessed type II resistance (resistance to fungal spread); however, other types of FHB resistance, such as resistance to DON accumulation, although more challenging to study, are also important. Moreover, most of the previous GWAS were performed based on single-locus models, such as the General Linear Model (GLM) and the Mixed Linear Model (MLM) (Bradbury et al., 2007). However, single-locus genome-wide association studies (SL-GWAS) are limited in detecting marginal effect quantitative trait nucleotides (QTNs) (Zhang et al., 2019b). Thus, many important loci associated with the target traits remain undiscovered because they do not satisfy the stringent criterion of the significance test. Current advances in multi-locus GWAS (ML-GWAS) models have improved the power and reliability of identifying causal loci for complex traits. to identify causal loci for complex traits. ML-GWAS also has a lower false-positive rate. It has been successfully applied to identify significant QTNs with subtle contributions to several agronomic traits in maize (Xu et al., 2018), rice (Misra et al., 2018), flax (Sertse et al., 2019), cotton (Li et al., 2018), and leaf rust in wheat (Fatima et al., 2020). At the same time, no studies have yet used ML-GWAS for FHB-related traits in durum wheat.

Thus, in the current study, we have analyzed a panel of Canadian and European durum wheat cultivars and breeding lines genotyped with the wheat 90K array and phenotyped for resistance to FHB to (1) determine the genetic architecture of FHB resistance, including resistance to DON accumulation, (2) test several GWAS models, including the SL and ML for FHB resistance-related traits, (3) identify potential candidate genes linked to the associated QTL, and (4) develop Kompetitive Allele-Specific PCR (KASP) markers from the QTL regions for utilization in plant breeding programs. In addition, we have addressed *Fusarium*-damaged kernels (FDK) and the relationship between FHB and quality traits such as protein content (PRO) and yellow pigment (YP). The results provide an insight into the complex genetic architecture of FHB resistance and reveal the QTL and genotypes of potential breeding value for FHB resistance. Additionally, the results should help better understand the genetic basis and diversity of DON accumulation in durum wheat and facilitate the reduction of DON contamination by stacking DON resistance QTL using marker-assisted selection (MAS).

## 2 Materials and methods

### 2.1 Plant materials

The germplasm used in this study consisted of 265 lines (**Supplementary Table S1**). This panel was primarily composed of durum from Canada, including elite Canadian and USA cultivars, advanced breeding lines, and recently developed germplasm from Canadian breeding programs (Crop Development Centre, University of Saskatchewan and Swift Current Research and Development Centre, Agriculture and Agri-food Canada) and research projects. The breeding lines and the cultivars were genotyped with *Rht-B1b* as per the previously published protocol (N’Diaye et al., 2017). The remaining lines were European *Triticum durum* cultivars and experimental lines developed by single seed descent by crossing a resistant tetraploid experimental line DBC-480 to Karur and Durobonus (susceptible European *T. durum* cultivars) and the advanced breeding line SZD1029K (**Supplementary Table S1**). These lines were developed and provided by the University of Natural Resources and Life Sciences, Vienna, Department of Agrobiotechnology, Institute of Biotechnology in Plant Production (IFA-Tulln), Austria, for this study. Karur and Durobonus are registered varieties in France and Austria, respectively. The DBC-480 line was developed at IFA-Tulln, Austria, by four generations of marker-assisted backcrossing of the highly resistant *Triticum aestivum* cultivar Sumai 3 into the background of the Austrian *T. durum* variety Semperdur and subjected to rigorous phenotypic selection for improved FHB resistance in field trials (Prat et al., 2017). The presence of the resistant *Fhb1* allele was verified using the SSR markers *Xgwm389, Xgwm533*, and *Xgwm493*. Cultivars Karur, Durobonus, and SZD1029K possess the semi-dwarfing allele *Rht-B1b*, while DBC-480 is a tall line harboring the *Rht-B1a* wild-type allele (Prat et al., 2017). Additionally, experimental lines descending from crosses of *T. durum* cultivars with moderately FHB-resistant *Triticum dicoccoum* and/or *Triticum dicoccoides* accessions from the IFA-Tulln, Austria, research program were also part of this study.

### 2.2 Phenotyping

Phenotypic data were obtained from multiple experiments conducted from 2019 to 2022 (inclusive) at FHB field nurseries (abbreviated afterward as FL) in Saskatoon (NSF), Saskatchewan, and Morden (MR), Manitoba, Canada, and in 2019 and 2020 in the University of Saskatchewan’s Crop Development Centre’s Greenhouse (GH), Saskatoon, SK (**Supplementary Table S11**). The infection recorded in 2021 was generally lower than normal because of extreme drought conditions (**Supplementary Figure S1**).

#### 2.2.1 Screening in the field FHB nurseries

At Saskatoon, the 265 lines were planted at the FHB nursery in hill plots with FHB susceptible and resistant checks in a randomized complete block design with three replications. For artificial inoculation, the inoculum was prepared by mixing equal amounts of spores from two virulent DON-producing *Fusarium graminearum* isolates, *Fg003* and *Fg004*, 3-*O*-acetyl-DON (3-AcDON) and 15-*O*-acetyl-DON (15-AcDON), respectively, originally collected from Saskatchewan. Aliquots of conidia stock solutions were stored at −20°C, then thawed at 37°C and diluted with distilled water to obtain the anticipated final spore concentration just before inoculation. Inoculations were performed when 50% of the plants in the earliest plot reached anthesis using a motor-driven backpack sprayer in the late afternoons. About 100 ml m^−2^ of conidial suspension at each inoculation cycle was sprayed onto the durum wheat heads. Inoculations were repeated at 2-day intervals and ended 2 days after the last plot flowered, resulting in up to six applications per plot. A sprinkler irrigation system provided adequate moisture after each inoculation to promote spore germination and fungal infection.

Agro-morphological traits, HD or anthesis (AD), days to maturity (MAT), and PH were assessed for all entries to determine their possible association with FHB traits. Heading date was recorded as the number of days from planting to the date when 50% of the heads in a plot had emerged, AD as days from planting until the first anther extruded out from the floret, and MAT when 50% of the plants reached physiological maturity. Plant height (cm) was measured as the distance from the base of the plant to the top of the spike, excluding awns. FHB incidence (INC) was the percentage of FHB-infected spikes/plots, and severity (SEV) was the percentage of symptomatic spikelets visually estimated 21 days after inoculation. At physiological maturity, 10 to 15 randomly infected spikes from each plot were collected and carefully threshed by hand to minimize the loss of FDKs. The grains were bulked, and a 10-g sample was milled in a Laboratory Mill 3610 grinder (2015 Perten Instruments^®^); 2 g subsample of flour was poured into a 15-ml polypropylene conical tube. DON quantification was performed using the high-throughput fast chromatography-tandem mass spectrometry (FC-MS/MS)-based method (Wang et al., 2022). LC-MS/MS conditions were developed on an Agilent Series 1260 Infinity HPLC system (Agilent Technologies, Mississauga, ON, Canada) coupled with an AB Sciex API 4000 hybrid triple quadrupole linear ion trap (QTRAP) mass spectrometer (AB Sciex, Concord, ON, Canada) equipped with a Turboionspray interface. Applied Biosystems/MDS Sciex Analyst software (Version 1.7) was used for system control and quantification. In addition, the FHB index (VRI) was calculated as (SEV × INC)/100 (Stack and McMullen, 1998), and INC-SEV-DON (ISD) index was calculated as (0.2 × INC) + (0.2 × SEV) + (0.6 × DON) following the protocol of the Prairie Recommending Committee for Wheat, Rye, and Triticale (PRCWRT, 2013).

The same lines were also evaluated at the Agriculture Agri-Food Canada (AAFC) FHB nursery near Morden, MB, in a randomized complete block design in a single 1-m-long row spaced 30 cm apart. *Fusarium graminearum* inoculum was prepared on corn kernels using four isolates: HSW-15-39 (3-ADON), HSW-15-87 (3-ADON), HSW-15-27 (15-ADON), and HSW-15-57 (15-ADON). Each isolate was inoculated in individual pans containing sterile corn and incubated for 1 month. After the corn was dried, it was stored in plastic tubs at 4°C until use. The inoculum was dispersed at a rate of 8 g per row, two times at weekly intervals, starting when the earliest lines were at the four- to five-leaf stage (Zadok’s stage 31) (Zadoks et al., 1974). The inoculum application was followed by irrigation three times a week (Monday, Wednesday, and Friday) using Cadman Irrigations Travellers with Briggs Booms. FHB incidence and severity were rated at 21 days post-anthesis. Full-row plots were harvested manually (avoiding the borders) and combined threshed. The harvested samples were sent to a service provider (Central Testing Laboratory Ltd., Winnipeg, MB) to perform FDK and DON analyses. In addition, the effect of DON accumulation on YP and PRO was investigated for the DON samples collected from the 2020 NSF using a near-infrared spectroscopy (NIR) analyzer.

#### 2.2.2 Screening in the greenhouse

Type II resistance to FHB was assessed in GH trials. Lines with check cultivars were planted in 1-gallon pots filled with a standard potting mix (type-3 soil). Six seeds per pot were sown, and after germination, only three plants were retained per pot for further growth and inoculation. Pots were designated as experimental units and arranged in a completely randomized design with two replications. Replicates were sown approximately 1–2 weeks apart, resulting in a 1–3-day difference in anthesis between replications. The temperature in the greenhouse was maintained at 22°C/18°C (day/night) with a 16-h photoperiod. The lines were inoculated with 3-ADON *F. graminearum* isolate SK-14-97 (obtained from the Cereal Pathology group, University of Saskatchewan, SK, Canada). First, a conidial suspension was plated onto potato dextrose agar media and exposed to permanent light for 4 to 7 days at 18°C. Then, a conidial suspension was adjusted to a concentration of 50,000 spores per mL using a hemocytometer. The anthesis date was recorded for each plant in a pot. Inoculations were performed at anthesis by pipetting 10 µl of conidia suspension between the lemma and palea of the two outer florets of one central spikelet per spike. A total of six heads per line was inoculated. The heads were then sprayed with sterile distilled water and covered with polyethylene transparent plastic bags for 24 hours to maintain high humidity. FHB symptoms were visually scored as the percentage of infected spikelets per spike at 21 days post-inoculation. At maturity, the inoculated heads of each line were harvested, combined from all replicates, threshed by hand to retain all the seeds, and ground into a fine powder for DON analysis following the procedure described in section 2.2.1.

### 2.3 Phenotypic data analyses

To obtain the best linear unbiased predictions (BLUPs) of the measured traits across test environments, the R package lme4 (version 3.4.2, https://www.r-project.org) was used for phenotypic data analysis using the following R-script: fitted to each genotype: Phenotype ∼ (1|Genotype) + (1|Repeat%in%Environment) + (1|Genotype&Environment). Lines were treated as a random factor, and BLUPs were estimated for all traits within and across environments. Broad-sense heritability (*H* ^2^) was then estimated using the variance components estimated from the previous equation. Pearson correlations between the BLUPs of the observed traits were calculated in R (Benesty et al., 2009).

### 2.4 Genotyping and SNP filtering

The mapping panel was genotyped with the 90K Illumina SNP chip to identify single nucleotide polymorphisms (SNPs). SNPs with minor allele frequencies <5% and missing data >10% were removed to avoid spurious marker–trait associations, leaving 13,504 SNPs for subsequent analyses (**Supplementary Table S10**). The physical positions of SNP markers were obtained from the latest Chinese Spring reference genome sequences (RefSeqv2.1: https://doi.org/10.1111/tpj.15289) to compare the QTL intervals with previous studies because most of them used this reference sequence.

#### 2.4.1 Genotyping with *Rht-B1b* marker

The *Rht-B1b* confers semi-dwarfism in durum (Peng et al., 1999). Therefore, we genotyped the lines with the *Rht-Blb* marker to see how this locus relates to FHB resistance. To compute the proportion of phenotypic variance explained by the marker *Rht-B1b*, we fitted a multiple linear regression model using *Rht-B1b* SNP as a predictor and FHB traits (mean FHB SEV and mean FHB INC) as a response. We also computed the proportion of mean PH variance explained by this marker for comparison. To implement the multiple linear regression model, we used the Ordinary Least Squares (OLS) regression function from the statsmodels library in Python 3.7.

### 2.5 Linkage disequilibrium and population structure

Linkage disequilibrium (LD) analysis was performed for each chromosome by computing *r* ^2^ values for all pairwise marker comparisons using the R genetics package (CRAN - Package genetics (r-project.org)). Marker positions were then used to investigate LD decay along each chromosome and across the entire genome. Background LD was estimated as the 90th percentile of the *r* ^2^ value of marker pairs on different chromosomes. LD decay distance was determined by fitting a non-linear model using the Hill and Weir method (Weir, 1979), with the *r* ^2^ threshold set at 0.2 and *r* ^2^ = half decay distance.

To define the appropriate number of subpopulations (*K*) for subsequent population structure and principal component analyses (PCA), the Bayesian information criterion (BIC) of each genotype was computed for 10 arbitrary clusters using the discriminant analysis of principal components (DAPC) function in the R package Adegenet 2.1.7 (Jombart, 2008; Jombart et al., 2020). Results were visualized into scree plots, and the number of clusters where the line trended horizontally was considered an appropriate number of ancestral subpopulations (*K*). The admixture proportion (ancestral coefficients) of genotypes was inferred based on sparse non-negative matrix factorization (sNMF) (Frichot et al., 2014) at the above (*K* + 1) estimated *K* as the number of subpopulations using the snmf function in R package LEA (Frichot and François, 2015). The admixture proportions of each genotype were summarized in bar plots using the plot function of the same package LEA. Principal component analysis was also performed based on the *K* subpopulation and the variation explained by each PC was computed. The clustering of the genotypes was visualized in a scatter plot based on the first two PCs. To confirm the population structure and the genetic relationships of the genotypes, phylogenetic analysis was performed following the Neighbor-Joining method using TASSEL 5 (Bradbury et al., 2007), and the web-based interactive tree of life (iTOL) (Letunic and Bork, 2016) was applied to visualize the trees.

### 2.6 Marker–trait association analysis

We applied seven ML and one SL GWAS method to capture loci underlying FHB and its related traits. The multi-locus methods include multi-locus random-SNP-effect MLM (mrMLM) (Wang et al., 2016), fast multi-locus random-SNP-effect EMMA (FASTmrEMMA) (Wen et al., 2017), Iterative Sure Independence Screening EM-Bayesian LASSO (ISIS EM-BLASSO) (Tamba et al., 2017), polygenic-background-control-based Kruskal–Wallis test plus empirical Bayes (pKWmEB) (Ren et al., 2018), fast mrMLM (FASTmrMLM) (Tamba and Zhang, 2018), and polygenic-background-control-based least angle regression plus empirical Bayes (pLARmEB) (Zhang et al., 2017), all implemented in the R package MrMLM v 5 (Wen et al., 2018). In addition, a haplotype (LD block) based on a restricted two-stage multi-locus multi-allele GWAS (RTM-GWAS) (He et al., 2017) was applied. The significant threshold for the first six in MrMLM was defined based on a logarithm of odds (LOD) >3, whereas for RTM GWAS Bonferroni corrected *p* < *α*/*n* was applied, where *α* = 0.05 and *n* = the number of LD blocks. For single-locus GWAS, a mixed linear model (MLM, with *Q* + *K* model) was applied using TASSEL 5 (Bradbury et al., 2007). The significant threshold of marker–trait association was set at a *p*-value adjusted based on Bonferroni correction (*α*/*n*, where *α* = 0.05 or significant threshold before multiple comparisons and *n* was the number of markers used for GWAS) (Sun et al., 2017). Population structure and kinship were included as correcting factors for all eight methods. Finally, significant SNP markers within one LD (13 Mbp) on the same chromosome were considered to represent a single locus. Quantile–Quantile (Q–Q) plots were generated to visualize the goodness of fitting for the GWAS model accounted for by the population structure and familial relatedness. The negative logarithm of the *p*-value from the model was calculated against the expected value based on the null hypothesis.

After identifying consistent and significant QTL regions, representative SNPs were extracted from the QTL regions and further investigated for combined additive effects for the traits SEV, INC, and DON. These combinations were tested with across environment BLUP values of the traits to assess which combination contributed to better resistance. Lines that carry resistant alleles at multiple loci were then selected and recommended for future breeding for FHB resistance based on the number of resistance alleles they contained.

### 2.7 Analysis of potential candidate genes for DON accumulation

To identify potential candidate genes within the interval of the DON QTNs captured by the GWAS analyses, the Chinese Spring reference wheat genome (RefSeqv2.1: https://doi.org/10.1111/tpj.15289) was used. Three highly significant MTAs for DON, *Ra_c4159_2716* (*QFhb-1A.1*), *Ra_c41921_951* (*QFhb-4B.4*), and *Ra_c29107_289* (*QFhb-6A*), were subjected to candidate gene analysis to identify genes/gene networks associated with resistance. LD analysis was performed using the R package gpart (Kim et al., 2019) by setting the coefficient of determination *r* ^2^ > 0.5 based on the genotype data and cv Chinese Spring’s gene coordinates. All polymorphic genes within the LD block (*r* ^2^ > 0.5) were assessed for their potential functional role based on information in different databases, including Knetminer wheat (https://knetminer.com/Triticum_aestivum/) and wheatgmap (http://www.wheatgmap.org/), orthologs of other species from Ensembl Plants (https://plants.ensembl.org/). To search orthologs in well-studied species such as rice and *Arabidopsis*, corresponding databases were used: the Rice Genome Annotation Project Database (RGAP) (http://rice.uga.edu/) and the Arabidopsis Information Resource (TAIR) (https://www.arabidopsis.org/), respectively. Genes with potential roles in FHB and related trait regulations were identified and summarized.

### 2.8 KASP marker development and analysis

The six FHB-related traits QTL that were consistently detected in multiple environments and by multiple GWAS models were chosen for KASP marker conversion. The most significant SNP from each of these six QTL was selected from the iSelect 90K Infinium array, and their probe sequences (Wang et al., 2014) were used for PCR primer development. Primers were developed from this probe sequence with two allele-specific forward primers and one common reverse primer (**Supplementary Table S9**). The KASP genotyping assays were performed on a CFX Touch Real-Time PCR Detection System (BioRad, Hercules, CA, USA) following the LGC Biosearch Technologies’ KASP genotyping manual (www.biosearchtech.com). Each KASP marker was verified on the GWAS panel to be congruent with the 90K iSelect genotyping scores. We then evaluated the genetic diversity of the SNPs significantly associated with the detected QTL in the Global Diversity Panel (GDP) of tetraploid wheat (Mazzucotelli et al., 2020). Six KASP markers, each representing a single locus detected by GWAS (see **KASP marker development and analysis**), were scored on the GDP, and data were recorded as “resistant” or “susceptible” alleles based on the marker effects estimated from the GWAS panel. Allelic frequencies of these loci were calculated separately in cultivars, landraces, domesticated, and wild emmer subsets of the GDP. Haplotypes were constructed based on the alleles at the six loci, and the number of accessions in GDP carrying each haplotype was summarized in a frequency bar plot. The top 10 most frequent haplotypes were georeferenced based on the coordinates of the country of origin of the accessions carrying the haplotypes and overlaid on the world map using the Quantum Geographic Information System (QGIS 3.8) (http://qgis.osgeo.org).

## 3 Results

### 3.1 Phenotypic variations

**Figure 1:**
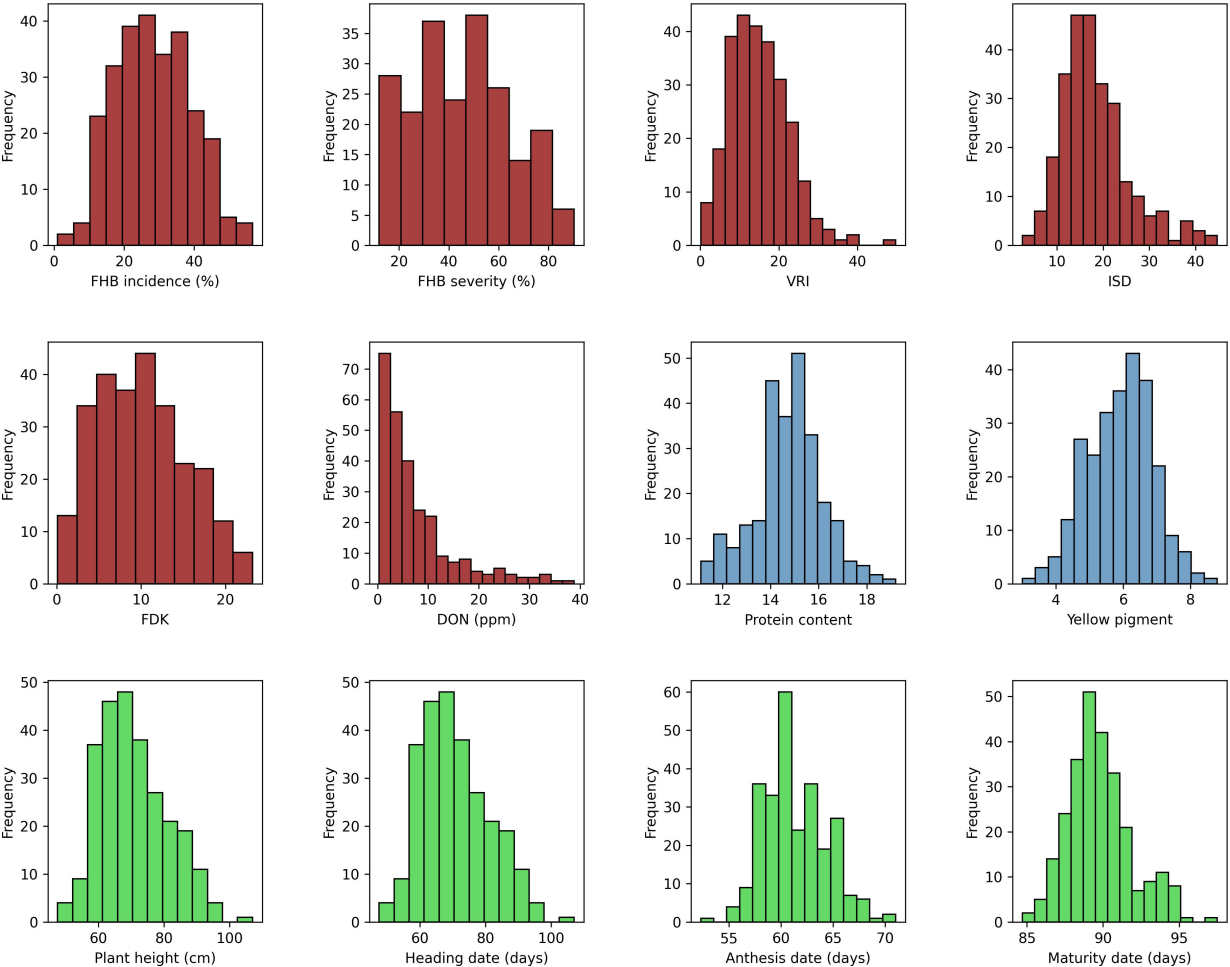
Phenotypic distribution of mean values for FHB incidence (INC; %), FHB severity (SEV; %), *Fusarium*-damaged kernels (FDK; %), deoxynivalenol concentration (DON; ppm), protein content (PRO; %), yellow pigment (YP, µg g^−1^), visual rating index (VRI; %), INC-SEV-DON index (ISD), plant height (PH, cm), heading date (HD, days), anthesis date (AD, days), and maturity date (MAT, days).

Descriptive statistics for all the traits tested under field and greenhouse conditions are presented in **Supplementary Table S2**. Analysis of variance of FHB traits showed significant (*p* < 0.001) variation among genotypes (data not shown). The phenotypic values ranged from 5% to 100% (GH) and 2% to 93% (FL) for SEV, 1% to 98% for INC, and 0.377 to 228.8 ppm (GH) and 0 to 97 ppm (FL) for DON. The mean values for all FHB traits were higher in 2020 at NSF, Saskatoon, than in the other environments, except where INC was higher in 2019 at the same location. In contrast, the lowest mean scores were recorded for 2021 at both field locations for all the traits. The frequency distribution of the mean values of the studied traits fit a normal distribution, except for DON (**Figure 1**). The broad-sense heritability (*H* ^2^, %) of the traits tested ranged from 38% (INC) to 93% (PH) (**Supplementary Table S2**).

**Figure 2:**
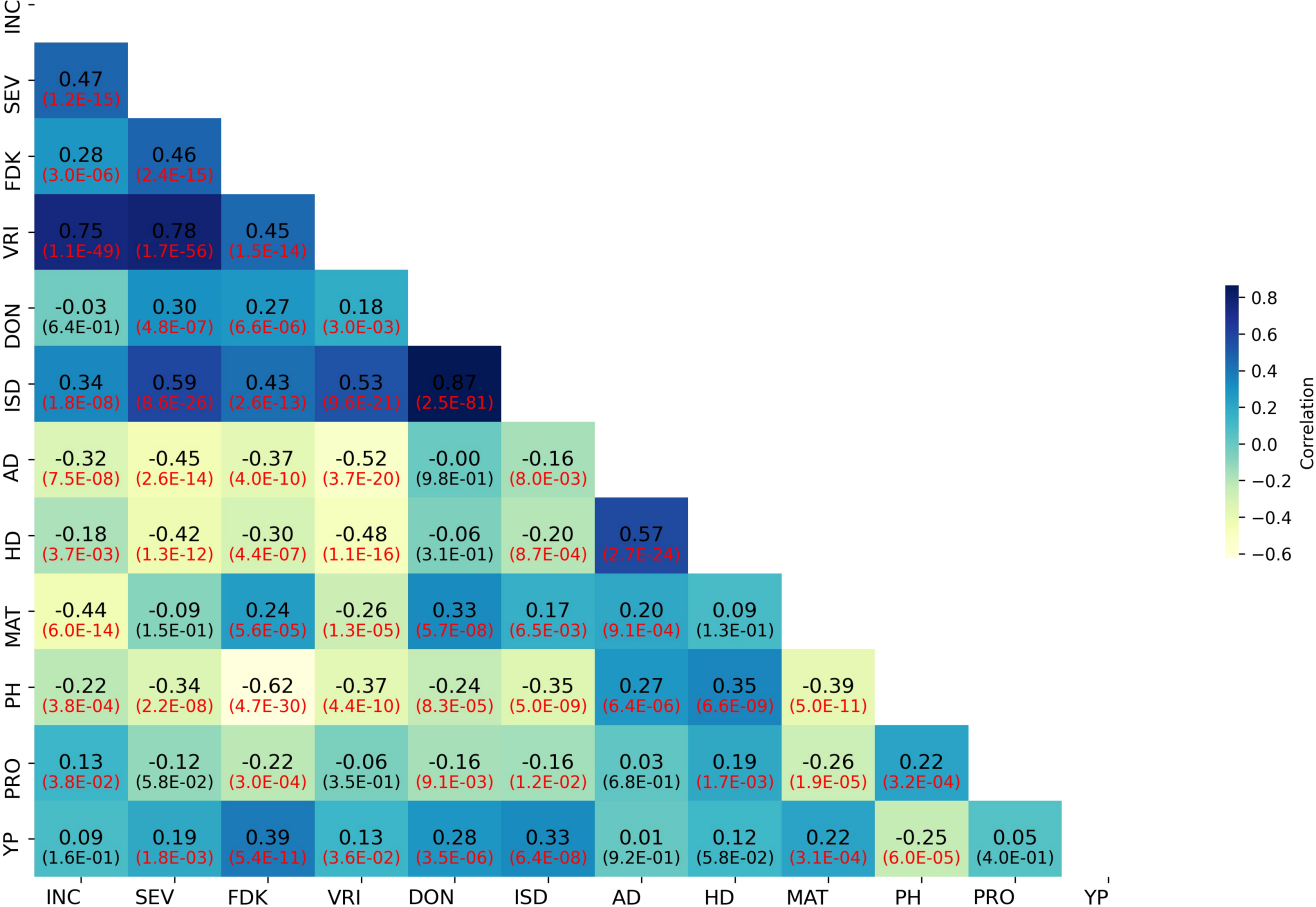
Correlation heatmap of FHB and agro-morphological trait BLUP values in the GWAS panel. INC, FHB incidence; SEV, FHB severity; FDK, *Fusarium*-damaged kernels; VRI, visual rating index; DON, deoxynivalenol; ISD, INC-SEV-DON index; AD, days to anthesis; HD, days to heading; MAT, days to maturity; PH, plant height; PRO, grain protein content; YP, yellow pigment.

Weak to strong correlations were detected between FHB SEV and INC and related traits. The correlations among BLUPs for FHB and morpho-physiological traits are presented in **Figure 2**. The correlation between BLUPs for SEV and INC (*r* = 0.47) was higher than that between SEV and DON (*r* = 0.30). BLUPs for DON were nearly equally correlated to SEV and FDK (*r* = 0.27). BLUPs for FDK correlated highest with SEV (*r* = 0.46). In contrast, BLUPs for PH, AD, and HD showed weak to moderate negative correlations with all FHB-related traits (**Figure 2**). The correlation coefficients of FHB response traits (SEV, INC, and DON) in all test environments ranged from 0.31 to 0.66 (**Supplementary Figure S2**). DON was highly correlated with SEV and FDK in individual environments (**Supplementary Figure S2**).

### 3.2 Population stratification

**Figure 3:**
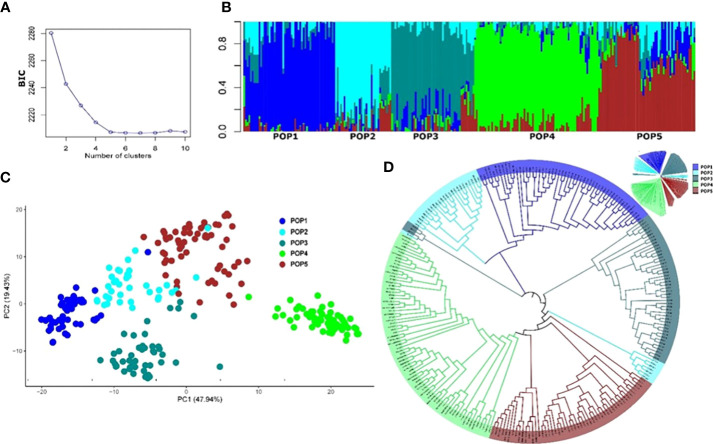
Population structure of the 265 durum wheat lines based on 13,504 SNP markers. **(A)** Cross-validation error showing the likely appropriate number of populations K to be 5. **(B)** Population structure based on genetic admixture for *K* = 5, where each bar represents a single line and the colored segments within each bar reflect the proportional contributions of each subgroup to that line. **(C)** Principal component analysis (PCA) plot of the first two principal components (PCs). Percentages in brackets indicate the variance explained by the PCs. **(D)** Topological view of the neighbor-joining phylogenetic tree.

The final number of SNP markers used for the analysis of population structure and GWAS analysis was 13,504. The appropriate number of subpopulations (*K*) was identified as five subgroups, essentially consistent with the breeding program of origin and pedigree (**Table S1**). The admixture, PCA, and phylogenetic analyses showed a similar pattern of clustering of individuals (**Figure 3**). Most of the experimental lines carrying the major resistance QTL *Fhb1* derived from Sumai 3 are clustered in Pop1, whereas some progenies from crosses with Sumai 3 *Fhb1* sources to European durum wheat cultivars (Prat et al., 2017) were clustered in Pop2 and Pop3. Pop4 comprised all the Canadian durum wheat cultivars and breeding lines, whereas Pop5 consisted of experimental lines derived from the intercrossing of cultivated lines with *T. turgidum* ssp*. dicoccum* and *T. turgidum* ssp*. dicoccoides* lines from the IFA-Tulln breeding program.

### 3.3 Marker-trait associations

For GWAS analysis, we performed and compared several single and multi-locus models. The single-locus MLM detected a total of 204 QTNs for FHB-related traits (INC, SEV, FDK, and DON), 245 QTNs for agro-morphological traits (PH, HD/AD, and MAT), and 31 QTNs for quality traits (YP and PRO) (**Supplementary Table S3**), based on individual environment and across environment BLUPs. For FHB resistance-related traits, the QTNs identified were distributed on all 14 chromosomes. For the MLM, the *Q*–*Q* plots illustrating the associations observed between SNPs and FHB resistance and related agro-morphological traits compared to expected associations after accounting for population structure and kinship relationships are presented in **Supplementary Figure S3**. The multi-locus models captured 288, 144, and 26 QTNs for FHB resistance, agro-morphological, and quality traits, respectively (**Supplementary Table S3**).

In total, 42 QTNs for FHB, 24 for agro-morphological, and six for quality traits were detected by two or more methods and explained greater than 15% of the phenotypic variation (**Supplementary Tables S4, S5**). Among the six ML-GWAS models used in our analysis, the ISIS EM-BLASSO model identified 93 QTNs, while FASTmrEMMA detected the lowest number of QTNs (37) for all the traits (**Supplementary Table S3**). We further grouped the 42 FHB QTNs into 31 QTL (**Supplementary Table S4**) based on the genome-wide LD of the panel (**Supplementary Figure S4**). Of these, *QFhb-1A.1*, *QFhb-2B.4*, *QFhb-3B.1*, *QFhb-3B.2 QFhb-4B.1*, *QFhb-5A*, *QFhb-6B.3*, and *QFhb-7B.2* were associated with QTNs identified by BLUP values and/or from GH screening (**Table 1**).

**Table 1:** Marker–trait associations detected by two or more GWAS models for FHB resistance and related traits using BLUP/field and GH data.

QTL	Representative SNP/sm	Chr	Marker position (Mbp)	Trait (environment)	LOD score	*R* ^2^ (%)	−log10(P)	MAF	Method
*QFhb-1A.1*	*Ra_c4159_2716*	1A	490.5	**SEV** (GH), **DON** (GH)	3.6–3.8	9.1–35.7	4.37–4.58	0.3	1, 6
*QFhb-2A.3*	*BS00000209_51*	2A	746.8	**DON** (BLUP), **SEV** (BLUP, GH)	3.3–6.1	23.2–24.0	4.05–6.95	0.32	3, 5, 6
*QFhb-2B.3*	*Excalibur_c39451_68*	2B	683.2	**SEV** (BLUP, GH)	4.3–6.4	12.1–16.9	5.02–7.28	0.47	1, 4, 5, 6
*QFhb-2B.4*	*Kukri_c12804_620*	2B	114	**DON** (GH), **SEV** (GH)	3.3–4.8	5.3–15.1	4.05–5.54	0.48	1, 4, 5, 6, R
QFhb-3A.1*	*RAC875_c4954_943 wsnp_Ex_c23633_32868822*	3A	9.0–13.0	**SEV** (20NSF), **ISD, PRO**	3.3–6.2	5.6–7.8	4.04–7.02	0.46	3, 5, 6
*QFhb-3B.1*	*TA004185-0427 RAC875_c5966_1854*	3B	3.2–9.9	**SEV** (BLUP), **DON** (BLUP), **ISD**	3.6–5.0	7.2–20.1	3.87–5.73	0.46	2, 4, 5, T
*QFhb-3B.2*	*RAC875_rep_c109105_57*, *Excalibur_c62826_254*	3B	578.2–578.8	**SEV** (BLUP), D**ON** (BLUP)**INC** (BLUP), **VRI**	3.2–11.9	10.5–36.9	4.64–12.84	0.37	1, 3, 4, 5, 6, R, T
*QFhb-3B.3*	*BobWhite_c6462_373*	3B	793.1	**SEV** (BLUP, GH)	3.4–5.6	13.1–24.9	4.08–6.41	0.36	1, 3, 4, 5
QFhb-4B.1^**^	*wsnp_BF482960B_Ta_1_4*, *RAC875_c27536_611*, *BS00021984_51*, *Ex_c101685_711*	4B	29.0–35.5	**SEV** (19NSF), **INC** (22MR), **DON** (21MR, 22MR), **FDK** (21MR), **PH**, **MAT**	3.8–20.1	5.3 – 52.3	4.52–21.23	0.29	1, 2, 3, 4, 5, 6, R, T
*QFhb-4B.3*	*Tdurum_contig14562_607*	4B	181.7	**INC** (BLUP)	3.9–5.2	10.1–11.3	4.64–6.03	0.38	3, 4, 6
*QFhb-4B.4*	*Ra_c41921_951*	4B	558.1	**DON** (BLUP), **ISD**	3.7–6.1	5.5–8.6	4.40–6.89	0.47	3, 4, 5, 6, R, T
QFhb-5A ^***^	*IAAV3365, BS00075959_51*, *wsnp_AJ612027A_Ta_2_5*, *BobWhite_c21949_150, wsnp_BF293620A_Ta_2_1*, *Kukri_c33022_198*	5A	586.6- 595.4	**INC** (19NSF, 20NSF, BLUP)**, SEV** (20NSF, 21NSF, BLUP)**, DON (**21MR, 22MR), **FDK** (21MR, 22MR), **VRI**, **HD, AD, HT, MAT**	3.3–25.0	5.9–44.5	3.74–28.49	0.40	1, 2, 3, 4, 5 6, R, T
*QFhb-5B.1*	*wsnp_Ra_c24619_34168104*	5B	508.8	**SEV** (BLUP, GH)	5.1–7.1	36.5–53.4	5.9–7.9	0.23	1, 4, 5, 6
*QFhb-5B.2*	*Ra_c2216_1442*	5B	591.1	**SEV** (GH), **FDK**	4.2–6.0	6.2–13.0	4.99–6.83	0.31	1, 4, 6
***QFhb-6A*** ^*^	*Ra_c29107_289, Excalibur_c25211_828*	6A	18.5–34.3	**DON** (GH),**INC** (BLUP), **MAT, PRO**	3.6-8.0	6.3–29.7	4.29- 14.85	0.42	1, 3, 4, 5, T
*QFhb-6B.1*	*Excalibur_c30648_924*, *Kukri_c3009_267*	6B	11.5–18.5	**SEV** (GH, BLUP), **DON** (BLUP), I**SD**	3.9–9.1	5.7–26.6	4.68–9.98	0.3	1, 2, 3, 4, 5 6, T
*QFhb-6B.3*	*Tdurum_contig45714_427*, *RAC875_c34994_183*	6B	123.8–128.7	**INC** (BLUP), **SEV** (BLUP), **DON** (GH, BLUP)	3.5–6.7	5.2–13.8	4.18–7.55	0.41	2, 3, 4, 5, 6
QFhb-7A^**^	*Tdurum_contig69067_405*	7A	662.0–671.0	**DON** (GH), **VRI,HD**	4.8–5.3	5.4–37.0	5.54–6.12	0.11	5, 6, T
QFhb-7B.1^**^	*Kukri_c51101_351*	7B	630.1	**DON** (GH), **SEV** (BLUP, GH), **HD, MAT**	3.4–6.0	11.2–24.1	4.14–6.79	0.45	4, 5, 6
QFhb-7B.2*	*Excalibur_c49736_1197*, *IAAV3713*	7B	706.9–728.7	**DON** (GH), **SEV** (GH, BLUP), **YP**	4.1–6.8	14.8–19.4	4.90–7.70	0.48	4, 5

Models: 1, MrMLM; 2, FASTmrEMMA; 3, pKWmEB; 4, ISIS EM-BLASSO; 5, FASTmrMLM; 6v, pLARmEB; R, TM and T, TASSEL.

Traits: SEV, FHB severity; INC; FHB incidence; DON, deoxynivalenol; FDK, Fusarium-damaged kernel; VRI, visual rating index (SEV * INC/100); ISD, INC-SEV-DON index (0.2 * SEV + 0.2 * INC + 0.6 * DON); HD, days to heading; AD, days to anthesis, MAT, days to maturity; PH, plant height; YP, yellow pigment; PRO, grain protein content; Chr, chromosome; MAF. minor allele frequency.

Location and year: NSF, North Sed Farm, Saskatoon, SK; MR, Morden, MB; GH, greenhouse; 19, 2019; 20, 2020; 21, 2021; 22, 2022.

QTL with ^*^ colocalized with PRO or YP, ^**^ colocalized with PH MAT and/or AD/HD, and ^***^ colocalized with MAT, PH, and AD/HD. Underlined QTL for these representable QTNs were converted to KASP markers.

**Figure 4:**
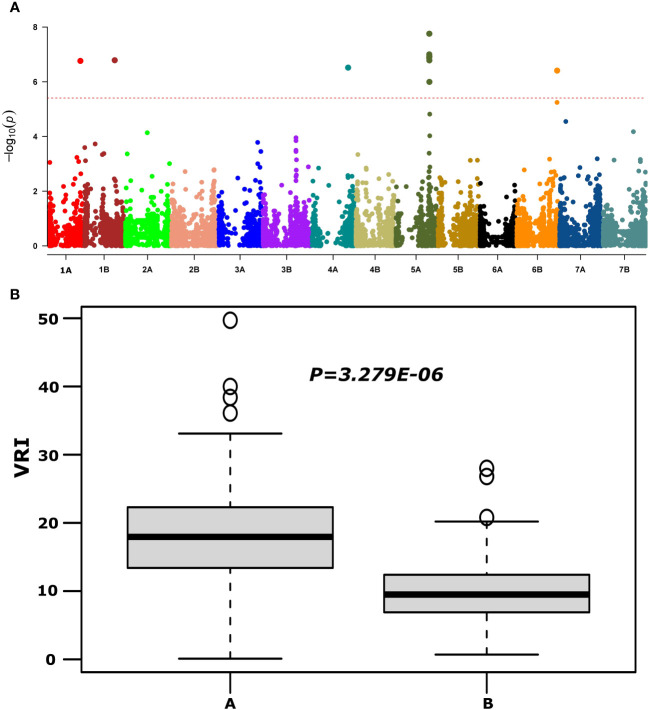
Manhattan plot reveals QTL for FHB visual rating index (VRI) using the MLM model (upper panel, **A**) and phenotypic variations at large effect QTL *QFhb-5A* (*IAAV3365*) for VRI (lower panel, **B**) based on overall mean.

QTNs for FHB SEV, INC, and DON that were detected in multiple environments with multiple models are presented in **Table 1** and **Supplementary Table S4**. Most agro-morphology-related trait-associated QTNs were consistent with the position of FHB QTL *QFhb-4B.1* and *QFhb-5A* (**Table 1**; **Supplementary Table S5**). *QFhb-3B.1* localized to a similar region of chromosome 3B as that previously reported for *Fhb1* (**Supplementary Figure S5**), which we confirmed by marker analysis (**Supplementary Table S1**). Contrast analysis revealed that, on average, lines carrying *Fhb1* showed reduced SEV, DON, VRI, and ISD (**Supplementary Figure S6**). QTL *QFhb-2A.4* and *QFhb-4B.4* were associated only with DON. Similarly, another QTL on chromosome 2B, *QFhb-2B.4*, was captured by the LD block approach (RTM GWAS) associated with DON with the highest −log10(P) value of 26.58 (**Supplementary Figure S7**). *QFhb-4B.1* and *QFhb-5A* were detected by eight of the GWAS models and associated with all FHB response and agro-morphological traits, followed by *QFhb-7B.2*, which was associated with YP as well (**Table 1**). *QFhb-4B.1* represents the most prominent genomic region for PH and MAT, with the highest LOD score (43.0) and phenotypic variance (60.3), followed by *QFhb-5A* with an LOD score of 25.0 and phenotypic variance of 44.5% for HD, AD, and MAT. *QFhb-6A* explained the maximum phenotypic variance (29.7%) for PRO (**Table 1**; **Supplementary Tables S4, S5**). As an example, the phenotypic variation for VRI based on the representative SNP at the most significant and consistent QTL, *QFhb-5A*, is presented in **Figure 4**, and the QTL region that spanned 586–595 Mbp on chromosome 5A is associated with multiple FHB resistance, and agro-morphological traits are shown in **Supplementary Figure S8**. Significance −log10(*p*-values) of the association of 13,504 SNPs based on RTM (LdBlock-based method) located on 14 chromosomes with BLUP values for DON, SEV, VRI, HD, MAT, and PH are depicted as Manhattan plot with multi-track Q–Q plots for each case (**Supplementary Figure S9**).

### 3.4 Candidate genes in the QTL regions for resistance to DON accumulation

The three loci strongly associated with DON, *Ra_c4159_2716 (QFhb-1A.1)*, *Ra_c41921_951 (QFhb-4B.4)*, and *Ra_c29107_289 (QFhb-6A)*, harbor candidate genes reported to be involved in disease resistance, defense, and stress responses, which were identified within their strong LD range of the associated genomic regions using Chinese Spring reference genome gene annotation (**Table 2**). The locus *Ra_c41921_951* on chromosome 4B associated with DON (FL) and detected by five different GWAS models harbors 11 genes, of which 10 were predicted to play a role in disease resistance; the remaining one is a stomatal opening gene. Two of the genes at this locus were predicted to contribute specifically to wheat stripe rust resistance. Of these, one was *TRAESCS4B02G280000*, which is an orthologue with genes encoding the SU (VAR)3-9 HOMOLOG 1 protein (SUVH1) that was assumed to regulate phenological traits such as days to flowering, heading, and maturity. Of the 11 genes, most (*n* = 8) were also predicted to regulate biological processes associated with drought resistance. The other GH_DON-associated locus on chromosome 6A, which was marked by *Ra_c29107_289*, harbors more than 20 genes that have been predicted to have a role in FHB resistance. This locus contains a higher density of genes (*n* = 13) that are orthologous with nitrate transporter 2.1 (NRT2.1). The QTL on chromosome 3B harbors *UDP-glucuronosyltransferases* (*UGTs*) gene families, which detoxify DON (**Table 2**).

**Table 2:** Predicted candidate genes detected within the LD block of selected significant QTL with *R* ^2^ > 15% for resistance to DON accumulation.

QTL	Representative SNP from LD block	Accession	Gene name	Chr	Start	Role
*QFhb-1A.1*	*Ra_c4159_2716*	TRAESCS1A02G295400	*TRAESCS1A02G295400*	1A	490418247	Harvest index
		TRAESCS1A02G295200	*UGP3*	1A	490306427	Harvest index
		TRAESCS1A02G294700	*TRAESCS1A02G294700*	1A	489600057	Plant height
		TRAESCS1A02G295300	*AMT2*	1A	490411509	Disease resistance, drought
		TRAESCS1A02G295600	*HSP70-9*	1A	490509793	Proline content
		TRAESCS1A02G294500	*TRAESCS1A02G294500*	1A	489357467	Stripe rust resistance
*QFhb-4B.4*	*Ra_c41921_951*	TRAESCS4B02G278100	*HSFA2D*	4B	560012594	Disease resistance, harvest index, salt tolerance
		TRAESCS4B02G278000	*ARD*	4B	560007429	Stomatal opening, stomatal resistance, turgor pressure, drought
		TRAESCS4B02G277900	*PME8*	4B	559854826	Stripe rust
		TRAESCS4B02G279000	*SEC15A*	4B	562354948	Disease resistance
		TRAESCS4B02G279100	*TRAESCS4B02G279100*	4B	562419106	Disease resistance, drought
		TRAESCS4B02G279300	*TRAESCS4B02G279300*	4B	562436091	Disease resistance, drought
		TRAESCS4B02G279200	*TRAESCS4B02G279200*	4B	562425162	Disease resistance, drought
		TRAESCS4B02G279500	*TRAESCS4B02G279500*	4B	562628791	Disease resistance, drought
		TRAESCS4B02G279400	*TRAESCS4B02G279400*	4B	562439286	Disease resistance, drought
		TRAESCS4B02G280000	*SUVH1*	4B	562880284	Stripe rust, day to heading/flowering, seed dormancy
		TRAESCS4B02G279600	*TRAESCS4B02G279600*	4B	562697710	Disease resistance, drought
*QFhb-6A*	*Ra_c29107_289*	TRAESCS6A02G037900	*SKIP4*	6A	18709035	Disease resistance, seed dormancy, self-incompatibility
		TRAESCS6A02G037800	*TRAESCS6A02G037800*	6A	18704699	Day to flowering, days to heading
		TRAESCS6A02G037600	*TRAESCS6A02G037600*	6A	18596060	Disease resistance
		TRAESCS6A02G029100	*MIK1*	6A	15435891	Disease resistance, drought
		TRAESCS6A02G029900	*CNL*	6A	15626844	Drought
		TRAESCS6A02G030100	*RGA5*	6A	15652828	Defense
		TRAESCS6A02G031200	*NRT2.1*	6A	15781020	Disease resistance, drought, Nitrate
		TRAESCS6A02G030900	*NRT2.1*	6A	15747526	Disease resistance, drought, Nitrate
		TRAESCS6A02G030700	*NRT2.1*	6A	15727844	Disease resistance, drought, Nitrate
		TRAESCS6A02G031000	*NRT2.1*	6A	15756560	Disease resistance, drought, Nitrate
		TRAESCS6A02G030800	*NRT2.1*	6A	15734520	Disease resistance, drought, Nitrate
		TRAESCS6A02G031100	*NRT2.1*	6A	15765759	Disease resistance, drought, Nitrate
		TRAESCS6A02G032000	*PIK6-NP*	6A	15916289	Defense
		TRAESCS6A02G034200	*BRM*	6A	16562153	Stripe rust, bacterial blight, day to flowering
		TRAESCS6A02G032400	*NRT2.1*	6A	15951566	Disease resistance, drought, nitrate
		TRAESCS6A02G032500	*NRT2.1*	6A	16098637	Disease resistance, drought, nitrate
		TRAESCS6A02G033800	*NQR*	6A	16462020	Oxidative stress
		TRAESCS6A02G033000	*NRT2.1*	6A	16386427	Disease resistance, drought, nitrate
		TRAESCS6A02G032800	*NRT2.1*	6A	16357746	Disease resistance, drought, nitrate
		TRAESCS6A02G033100	*NRT2.1*	6A	16398961	Disease resistance, drought, nitrate
		TRAESCS6A02G032900	*NRT2.1*	6A	16374353	Disease resistance, drought, nitrate
		TRAESCS6A02G033200	*NRT2.1*	6A	16408185	Disease resistance, drought, nitrate
		TRAESCS6A02G032600	*PRP39*	6A	16226818	Day to maturity
		TRAESCS6A02G033400	*HIDM*	6A	16443279	Disease resistance, drought

### 3.5 KASP markers for the FHB-associated QTL

**Figure 5:**
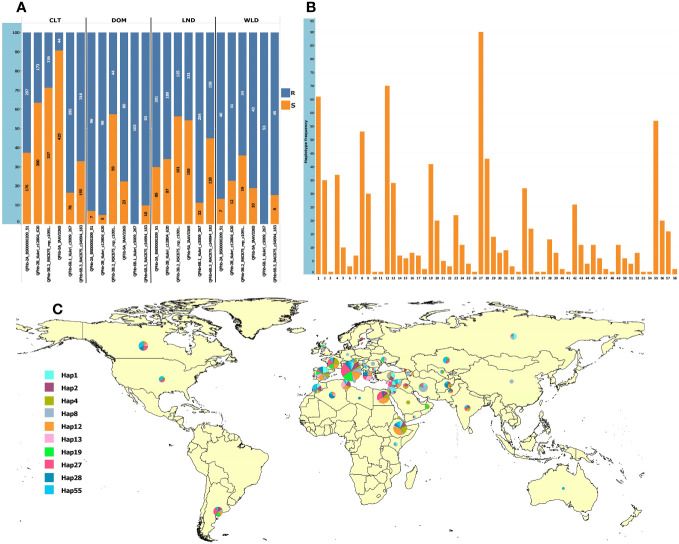
Allelic frequencies in the GDP panel and the geographic distribution of the 10 most frequent haplotypes. **(A)** Allelic frequencies of the six representative SNPs from the most consistent QTL in cultivars (CLT), domesticated emmer (DOM), landraces (LND), and wild emmer (WLD); the *y*-axis shows the percentage, and the labels show the absolute frequencies. **(B)** Haplotype frequencies based on the six loci. **(C)** Geographic distribution of the top 10 most frequent haplotypes. The haplotype pie chart circle size is proportional to the number of accessions from the corresponding country.

For MAS, we converted to KASP marker QTN representing six QTL that were (a) detected in multiple environments and (b) by multiple GWAS models (underlined QTL in **Table 1**). The markers were robust and clearly clustered lines into two discrete allelic groups (**Supplementary Figure S12**). These were then scored in the GWAS panel and a subset of the GDP and compared with available 90K data (**Supplementary Table S9**). The KASP markers developed from *Kukri_c12804_620* (*QFhb-2B.4*) scored two loci, one of which was much more congruent with the 90K data associated with the FHB resistance QTL, thus was used for further analysis (**Supplementary Table S9**). Analysis of the SNP markers in the GDP panel revealed that the frequency of FHB resistance alleles among the GDP wheat groups (cultivar, landrace, domesticated, and wild emmer) was highly variable, especially for *QFhb-5A* (**Supplementary Figure 6A**). Surprisingly, the frequencies of resistance alleles were low in cultivars (**Figure 5**; **Supplementary Table S8**). In contrast, wild and domesticated emmers present in the GDP all carried the resistance allele at *QFhb-6B.1* (**Figure 5A**; **Supplementary Table S8**). Next, we grouped the alleles from each QTL into 58 haplotypes and identified their frequency within the GDP (**Supplementary Figure 6B**). The majority of the 10 most common resistant haplotypes were concentrated in accessions collected from the Mediterranean and Middle East countries (**Figure 5C**). The haplotype ID and frequencies for the selected six markers are presented in **Supplementary Table S7**.

## 4 Discussion

### 4.1 Trait heritability and correlation

This study identified QTL as having a major effect on DON accumulation based on both field and GH data, followed by SEV and INC. The heritability for DON was 86%, higher than for SEV (61%) and INC (38%). This is likely because we used the high-throughput fast chromatography-tandem mass spectrometry (FC-MS/MS) method, which is not subject to the same error rate as the visual scoring of FHB incidence and severity in the field (Wang et al., 2022). Similarly, the heritability for SEV was higher in the GH (72%) than in the field (61%). Conversely, He et al. (2016b) found low heritability estimates (0.34) for FHB traits in a point-inoculated trial. This could be because of genotypic differentiation that occurred due to point (GH) and spray (FL) inoculation, where some genotypes reacted differently to the two methods, resulting in a large interaction variance. According to Miedaner et al. (2003) and Schroeder et al. (1963), genotypes that are resistant to spray but susceptible to point inoculation should have type I resistance (resistance to initial infection), whereas genotypes susceptible to spray but resistant to point inoculation should possess type II resistance. Therefore, the QTL that were significant in two or more environments for FHB response should be emphasized for resistance breeding.

The negative correlations between FHB resistance and PH and HD/AD also agreed with previous findings summarized by (Prat et al., 2017; Steiner et al., 2017; Haile et al., 2018). The relationship between HD and PH with FHB was moderate and negative (**Figure 2**; **Supplementary Figure S10**), unlike the strong correlation reported by Ruan et al. (2020). Correlations between HD/AD and FHB may have a confounding effect on the association mapping results because these two traits can cause infection escape, and weather conditions such as humidity and temperature during anthesis can significantly affect the success of FHB infection. Therefore, it is vital to score FHB following the HD/AD days for individual germplasm (as used in this study) or correct for these factors in individual nurseries (Nannuru et al., 2022).

DON production plays a significant role in the spread of FHB within a spike (Bai et al., 2002); however, according to Bai et al. (2002), DON production is not necessary for initial infection by the fungus. The correlation between FHB SEV and DON accumulation in the present study was positive, with a coefficient of 0.43 (**Figure 2**). Reviews by Buerstmayr and Lemmens (2015) and Lemmens et al. (2016) also indicated a positive correlation between FHB severity traits and mycotoxins. However, He et al. (2014) found a negative relationship between FHB index and DON concentration, whereas Bai et al. (2002) reported a complicated relationship between SEV and DON, i.e., cultivars moderately resistant and moderately susceptible to FHB SEV usually had higher DON accumulation than resistant cultivars, but there also were exceptions, especially for cultivars with moderate resistance.

### 4.2 Marker–trait associations

Depending on the type of trait, applying appropriate model/s and statistical method/s is crucial for reliable results in GWAS. FHB resistance is a polygenic and multifactorial complex trait controlled by multiple small-effect loci. Multi-locus methods are more effective and efficient in capturing such small effect loci than single-locus models (Segura et al., 2012; Xu et al., 2018; Zhang et al., 2019a). However, combining multi- and single-locus methods is recommended to increase detection power and robustness (Li et al., 2018). It is also advantageous to integrate multiple GWAS methods to cross-check results to improve QTL confidence (Zhang et al., 2020).

Previous mapping studies have identified QTL for FHB resistance with minor to moderate effects that were repeatedly detected on 11 of the chromosomes of tetraploid wheat (Otto et al., 2002; Gladysz et al., 2007; Kumar et al., 2007; Ghavami et al., 2011; Buerstmayr et al., 2012; Ruan et al., 2012; Buerstmayr et al., 2013; Miedaner et al., 2017; Ruan et al., 2020). In our study, we identified QTN on all 14 chromosomes. Only minimal variation for resistance to FHB has been reported in cultivated durum wheat (*Triticum turgidum* ssp*. durum*); therefore, introgressions of resistance from its relatives (e.g., *T. turgidum* ssp*. dicoccum, T. turgidum* ssp*. dicoccoides*) are a priority and have shown promise, particularly in conferring resistance to FHB SEV (Buerstmayr et al., 2013). In this study, we identified QTN regions, *QFhb-1A.1*, *QFhb-2A.3*, *QFhb-6A*, and *QFhb-7B.2*, with significant contributions to the phenotypic variation that were identified from exotic sources. Several other studies also identified the main effect of QTL from exotic genetic resources, such as *T. carthlicum* (Sari et al., 2018), *T. turgidum* ssp. *dicoccoides* (Otto et al., 2002; Kumar et al., 2007; Ruan et al., 2012; Buerstmayr et al., 2013), and *T. turgidum* ssp*. dicoccum* (Buerstmayr et al., 2012; Zhang et al., 2014), stressing the importance of these germplasm collections as sources of FHB resistance genes to support breeding. The majority of the QTL detected by Ruan et al. (2020), were confirmed in this study, but the QTL effects were higher in this study. One potential explanation is that we tested a larger panel (265 lines) in five field environments and twice in the greenhouse, which may contribute to increased genetic variance and heritability expression.

#### 4.2.1 QTL associated with multiple FHB resistance traits

Responses to different FHB resistance types are generally correlated, and complex biological and physiological mechanisms are involved in the coordination of their expression. Seven of the significant and stable QTL regions were detected for FHB resistance, *QFhb-1A.1*, *QFhb-2B.4*, *QFhb-3B.1*, *QFhb-3B.2*, *Fhb-4B.1*, *QFhb-5A*, and *QFhb-6B.3*, which affect SEV, INC, and DON. Moreover, *QFhb-3A.1* and *QFhb-7B.2* were associated with PRO and YP, respectively, in addition to SEV and DON. These QTL were of major importance based on consistency across models, their detection across multiple testing environments, and their association with agro-morphological traits like plant height and heading time.

Due to the colocalization of QTL, *QFhb-4B.1*, and *QFhb-5A* with PH and other agro-morphological traits, respectively, we discuss them independently. Simultaneously, we compared the locations of the QTL regions in this study with those of previous studies. For some of the QTL, comparisons across different findings were difficult due to the differences in the marker platforms and the lack of reliability in chromosome assignment among existing consensus maps.

##### 4.2.1.1 Chromosome 1A

The QTL *QFhb-1A.1* contributed 35.1% to the phenotypic variance for DON in greenhouse (GH) trials. While we did not detect its effect in field trials, this QTL is coincident with minor QTL for FHB INC, and SEV in Canadian tetraploid germplasm (Ruan et al., 2020), Chinese elite germplasm (Wu et al., 2019), and US soft red winter wheat (Gaire et al., 2021). Our candidate gene analysis revealed that this QTL colocalizes with an ammonium transporter gene (AMT2), a family of proteins transporting ammonium salt and its analogs (**Table 2**). Several studies have reported that ammonium influences the interaction between plants and pathogens (Jiang et al., 2019); thus, this gene might influence the pathogen’s ability to produce DON.

##### 4.2.1.2 Chromosome 2A

Among the QTL*, QFhb-2A.3* was consistent for FHB SEV, as it was detected in five of the testing environments and using across-environment BLUP values. In the same physical region of *QFhb-2A.3*, Gladysz et al. (2007) identified a QTL for resistance to FHB severity derived from the resistant *T. turgidum* ssp*. dicoccoides* accession Mt. Hermon#22 and a minor QTL from a diverse tetraploid population reported by Ruan et al. (2020) in the 762–769 Mb interval. Ruan et al. (2020) reported a QTL 16 Mb distant from *QFhb-2A.3*, whereas Steiner et al. (2019b) reported a QTL for FHB resistance in the same region. The physical positions of all the loci identified on chromosome 2A were physically distant from the photoperiod gene *Ppd-A1*, a major gene responsible for flowering time in wheat (Distelfeld et al., 2009). This is fortunate because these QTL are not being influenced by AD or HD, both of which could potentially limit their use in breeding programs.

##### 4.2.1.3 Chromosome 3B

The two 3B QTL, *QFhb-3B.1* and *QFhb-3B.2*, were associated with all FHB response traits in this study. *QFhb-3B.1* was identified in a similar region as the 3B QTL identified in most previous studies (Anderson et al., 2001; Liu et al., 2006; Buerstmayr et al., 2009; Arruda et al., 2016; Steiner et al., 2019b; Ruan et al., 2020; Nannuru et al., 2022). This region also corresponds to the physical interval of the major FHB QTL, *Fhb1*, located on 3BS around 7.6–13.9 Mb (Anderson et al., 2001; Liu et al., 2006). This was expected as durum wheat lines that carry *Fhb1* introgressed from Sumai 3 (Prat et al., 2017) were included in our diversity panel, and most showed the favorable allele for this locus. Tetraploid landrace, TG3487, with mean FHB SEV 6.2% (**Supplementary Table S11**), also carried the favorable loci for *QFhb-3B.1* (**Supplementary Table S1**). *Fhb1* is well known for conferring resistance to FHB severity (Anderson et al., 2001; Buerstmayr et al., 2003; Cuthbert et al., 2006)). In this study, we also confirmed the importance of *QFhb-3B.1* to reduced DON accumulation in tetraploid wheat. Indeed, resistance to SEV conferred by *Fhb1* is associated with its ability to inactivate DON (Schweiger et al., 2016). Similarly, *QFhb-3B.2* is a pleiotropic locus associated with SEV, INC, DON, and VRI and contributed up to 36% of the phenotypic variance. It was considered a novel region, as we are not aware of previous studies reporting this region. *UDP-glucuronosyltransferases* (*UGTs*) reside in this QTL region; they are DON-responsive genes potentially involved in DON detoxification, are induced by *F. graminearum* and enhance resistance to FHB in wheat (Zhu et al., 2020; Wu et al., 2022). Plants can use UGTs to chemically modify DON to produce DON-3-glucosides, which are less toxic than DON (Jia et al., 2009). Accordingly, wheat could induce UGTs to respond to infection and detoxify the trichothecene mycotoxins. Interestingly, UGT genes had higher expression levels in FHB-resistant wheat genotypes such as “Sumai 3” (Gottwald et al., 2012).

##### 4.2.1.4 Chromosome 5B

QTL *QFhb-5B.1* conferred up to 53% of the phenotypic variation for FHB SEV and DON and was detected around 508.8 Mb, 114.2 Mb away from the vernalization gene *VRN-B1* (613.0 Mb). It was not associated with agro-morphological traits. Ruan et al. (2020) identified a QTL for FHB INC and FHB index in the *VRN-B1* region (577–694 Mb) that affected HD, supporting that this QTL is indeed unique from that published previously.

##### 4.2.1.5 Chromosome 7B

On 7BL, *QFhb-7B.2* was associated with FHB SEV and DON. The allele associated with reduced SEV and DON was also associated with elevated YP, an important end-use quality trait in durum wheat (Pozniak et al., 2007). The association with YP was perhaps not surprising since *QFhb-7B.2* is physically located near *Psy1-B1*, a critical gene in the carotenoid biosynthetic pathway that is partially responsible for the elevated yellow color in the grains of durum wheat (Pozniak et al., 2007). We did notice, however, that this QTL was also associated with a slight reduction in grain protein content. Indeed, DON showed a weak negative correlation with PRO (−0.17) (**Figure 2**). This is in contrast to previous reports that observed a positive correlation between FHB symptoms and PRO, likely because of the degradation of starchy content by *Fusarium* spp. and of proteins by the hyphae of the fungus causes the consequent increase in PRO (Boyacioglu et al., 1992). However, given the relatively small negative correlation, any potential loss in PRO is likely to be overcome in breeding programs by simultaneous selection for low DON and higher PRO, as has been done for other negatively correlated traits (Ruan et al., 2021).

#### 4.2.2 QTL regions for FHB response colocalized with agro-morphological traits

Responses to FHB resistance involve complex biological mechanisms and environmental conditions. Wheat is most susceptible to FHB infection during anthesis, particularly if the flowering period coincides with warm, humid conditions that promote disease development (Hooker et al., 2002). For example, HD (or AD) might affect the disease scores when the germplasm differs in maturity, and correlations can be positive or negative, depending on weather conditions in different years. Consequently, FHB traits and flowering time QTL are often associated with mapping studies (Sari et al., 2019; Ruan et al., 2020). In our study, two of the major QTL for FHB responses, *QFhb-4B.1* and *QFhb-5A*, colocalized with all agro-morphological traits (**Table 1**). In addition, *QFhb-2B.1*, *QFhb-6A, QFhb-7A*, and *QFhb-7B.1* colocalized with minor PH and/or HD and MAT QTL. So, while these QTL may be useful for targeted selection breeding, their selection may result in undesirable influence on phenotypic expression of flowering time, time to maturity, and plant height. Indeed, taller plants have a greater chance to escape infection, and increased height likely reduces the relative humidity near the wheat spikes, but an excessive plant height is undesirable in commercial durum wheat production.

##### 4.2.2.1 Chromosome 4B

Five QTL regions were identified on Chr 4B (**Table 1**; **Supplementary Tables S4**) in our study. Of these, QTL *QFhb-4B.4* was solely associated with DON and ISD, an index based on 60% of the DON value (PRCWRT, 2013). In the same interval, a QTL for FHB SEV was previously identified (Nannuru et al., 2022). *QFhb-4B.1* (spanning 29.0–35.5 Mb physical interval) was associated with all FHB response traits, including DON. However, this QTL consistently colocalized with major QTL (*R* ^2 = ^60.3 and LOD = 43.0) for PH and MAT. In a similar QTL region, Nannuru et al. (2022) identified QTL for resistance to FHB severity and DON in the spring European wheat panel using scorings corrected for the confounding effect of PH and HD. Plant height has been reported to be associated with FHB (Mao et al., 2010; Miedaner et al., 2017; Sari et al., 2018; Steiner et al., 2019b; Buerstmayr and Buerstmayr, 2022). The correlation analysis in the present study further confirms these reports, as FHB SEV, INC, and DON were negatively correlated with PH.

Based on our analysis, *QFhb-4B.1* localizes to a similar genomic interval as *Rht1* (*Rht-B1*) on chromosome 4B. We confirmed this by assaying the allelic state of *Rht-B1* using a robust DNA marker for that gene (Ellis et al., 2002) (**Supplementary Table S1**). In our diversity panel, the *Rht-B1b* marker explained 4.1% and 5.7% of the phenotypic variance of the mean FHB SEV and INC, respectively, whereas it explained 49.3% of the mean PH variance. According to previous reports, there is an association between *Rht-B1a* (GA-sensitive allele), low FHB infection, and tall plant height (He et al., 2016a; Dhariwal et al., 2020; Nannuru et al., 2022). Given the strong influence of *Rht-B1b* on FHB susceptibility in our population, other dwarfing genes that do not impact the phenotypic expression of resistance should be considered by breeders. One GA-insensitive dwarfing gene, *Rht24*, may be a suitable alternative because it reduces plant height without influencing FHB (Herter et al., 2018).

##### 4.2.2.2 Chromosome 5A

One of the most stable QTL regions identified in this study was *QFhb-5A* (**Supplementary Figure S8**), which spans 586.6–595.4 Mb and was associated with all the FHB response traits considered in the current study. However, this QTL also colocated within the interval of major QTL (*R* ^2 = ^44.5) for HD/AD and MAT. The physical position of this QTL supports that it is not *Fhb5* (Buerstmayr et al., 2009; Xue et al., 2011), which spans a physical interval between 105.4 and 214.2 Mb of chromosome 5A (Ma et al., 2020). However, *QFhb-5A* overlaps with other QTL reported previously—5A2 (Sari et al., 2020) with similar representative SNPs *wsnp_AJ612027A_Ta_2_5* and *Kukri_c33022_198*, spanning a physical interval of 550.5–556.8 Mb (Sari et al., 2018) and 551.0–556.6 Mb (Sari et al., 2020). Similarly, Ruan et al. (2020) reported a QTL for FHB response and flowering time in the same physical interval (585–591 Mb). *QFhb-5A* also appears to be congruent with a 5A QTL for FHB resistance derived from European winter wheat cultivars Arina, Pirat, and Apache (Gervais et al., 2003; Paillard et al., 2004; Draeger et al., 2007; Holzapfel et al., 2008; Gaire et al., 2021) and in Chinese spring wheat (Zhu et al., 2020) and European spring wheat (Nannuru et al., 2022).

Flowering time is an adaptive trait of wheat, and it is regulated by a complex pathway to ensure that grain filling occurs under favorable conditions (Royo et al., 2020). Allelic variation at the *Vrn1* loci in wheat regulates flowering time, plant height, spike, and spikelet morphology (Li et al., 2019). We determined that *Vrn-A1* correlates with the physical interval of *QFhb-5A* and has been associated with FHB response previously. A similar result is reported by Gervais et al. (2003); He et al. (2016b), and Klahr et al. (2007), where the QTL for the FHB response was likely to be conferred by the pleiotropic effects of *Vrn-A1*. According to He et al. (2016b), the effect of *Vrn-A1* on FHB resistance decreased substantially when DH was used as a covariate. Thus, current evidence supports that *QFhb-5A* is most likely pleiotropically associated with the effect of *Vrn-A1* on flowering time, despite this gene only having a moderate influence on flowering time in durum wheat (Ruan et al., 2020). However, as noted prior, *Vrn-A1* does not influence flowering time alone but also regulates spike and spikelet development, and plant height. Our current hypothesis is that *QFhb-5A* is not associated with HD per se but is likely associated with the pleiotropic effects on spikelet formation. Indeed, spike development impacts type II (resistance to FHB spread in the wheat spike), and mutants of *Vrn1* in wheat delay the formation of terminal spikelets and increase the overall number of spikelets per spike (Li et al., 2019). Therefore, caution should be taken when using this QTL in resistance breeding because the mechanism of colocalization between the vernalization genes and FHB resistance is still unclear.

### 4.3 Exploitation of FHB resistance QTL in durum wheat breeding

We identified *QFhb-3B.1* in our diversity panel as an effective QTL to reduce INC and SEV (**Supplementary Figure S6**), and based on marker data, we confirmed this QTL is congruent with *Fhb1* (**Supplementary Table S1**). *Fhb1* is the well-studied QTL for FHB resistance breeding in wheat and provides type II resistance and the ability to detoxify DON (Lemmens et al., 2005). However, the low frequency of resistance alleles in elite wheat breeding parents and concerns about the detrimental effect of linkage drag have limited the utilization of *Fhb1* in breeding programs (Brar et al., 2019). In addition, the introgression of *Fhb1* into durum wheat has been challenging due to unstable expression in a durum genetic background (Zhao et al., 2018). However, Prat et al. (2017) successfully introgressed *Fhb1* from Sumai 3 into two European durum cultivars (Karur and Durobonus) and an Austrian breeding line (SZD1029K), which we included in our diversity panel. We also included several breeding lines in our panel carrying *Fhb1* that are derived from introgression efforts (**Supplementary Table S1**). Allelic variation at *Fhb1* was associated with all FHB-related traits except for DON (**Supplementary Figure S6**), confirming the effectiveness of *Fhb1* introgression in some durum backgrounds. Moreover, we identified additional lines in our diversity panel that appear to be carriers of *Fhb1* based on KASP marker (*Fhb1-TaHRC*) data (**Supplementary Table S1**). However, the effect of *Fhb1* varied depending on the durum genetic background and the individual environments, explaining between 7% and 20% of the phenotypic variance, and indeed, some accessions harboring *Fhb1* showed moderate susceptibility to FHB in our studies. Previous studies have also demonstrated that *Fhb1* is neutral or does not effectively increase resistance to FHB alone in certain genotypes (Pumphrey et al., 2007). The discrepancies observed among *Fhb1* introgressions with different durum backgrounds may be due to differences in their respective genetic resistance architectures (Prat et al., 2017) or the presence of susceptibility factors and suppressor genes in its genome (Ghavami et al., 2011) that compromised the expression of FHB resistance in durum wheat. Furthermore, we noted that *Fhb1* introgressions showed reduced YP and PRO compared to non-carriers (**Supplementary Figure S11**).

In Canada, durum wheat cultivars such as Brigade (Clarke et al., 2009), Transcend (Singh et al., 2012), CDC Credence (Pozniak et al., 2020a), CDC Defy (Pozniak et al., 2020b), and AAC Schrader show improved FHB resistance relative to other elite durum wheat cultivars in Canadian production. These were developed by accumulating native resistance genes (Ruan et al., 2020; Sari et al., 2020) through phenotypic selection. Several Canadian durum wheat cultivars and advanced breeding lines—for example, Brigade, DT1021, DT696, Transcend, and DT2004—carry four favorable alleles for reduced DON accumulation. Therefore, these cultivars and breeding lines are a useful platform for stacking additional FHB resistance QTL (including *Fhb1*), which should result in further improvements in FHB resistance.

**Figure 6:**
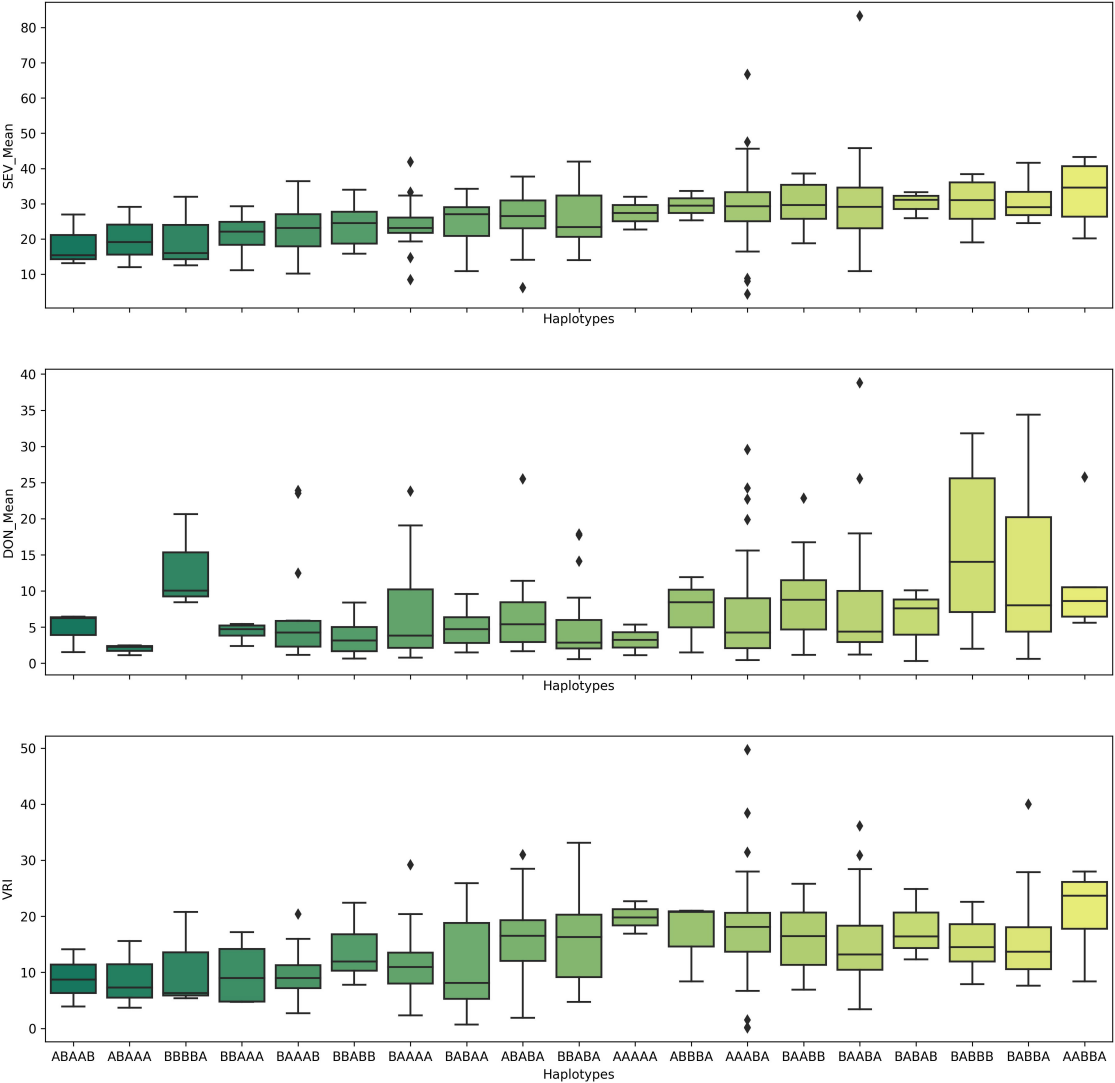
Pyramiding effects of *QFhb-2A.3*, *QFhb-3B.2*, *QFhb-6A*, *QFhb-6B.1*, and *QFhb-7B.2* provide resistance to FHB SEV, INC, and DON accumulation. SEV, severity (%); INC, incidence (%); DON, deoxynivalenol (ppm).

Because the QTL *QFhb-2A.3*, *QFhb-2B.4*, *QFhb-3B.2*, *QFhb-6B.1*, and *QFhb-6B.3* were not associated with the agro-morphological traits, these are likely a higher priority for immediate use in durum wheat breeding programs. Lines with resistance alleles at these QTL (ABAAA haplotype, **Figure 6**) showed relatively less mean FHB VRI and DON accumulation, whereas those lacking the resistance alleles showed high FHB SEV and more DON accumulation (**Figure 6**). Therefore, these lines carrying favorable haplotypes (such as ABAAA) could be good sources for FHB resistance breeding.

The GDP is an internationally established diversity panel comprising a wide representation of *Triticum turgidum* ssp. *durum* cultivars, modern germplasm, and landraces, along with a selection of emmer (*T. turgidum* ssp. *dicoccum*, *T. turgidum* ssp. *dicoccoides*) and primitive tetraploid wheats (Mazzucotelli et al., 2020). The panel is publicly available and is recognized by the durum wheat community as a vehicle to drive the discovery of useful alleles and their immediate deployment in breeding activities. To that end, we have developed KASP markers for utility in breeding programs (**Supplementary Figure S12**; **Supplementary Table S9**) and used these to characterize the six consistently expressed FHB-trait-associated QTL (see results section 3.5) and to describe their haplotypes in the GDP (**Supplementary Table S9**). This analysis revealed a relatively low frequency of resistance-associated haplotypes for some of the QTL in cultivated durum wheat, while a higher frequency of resistance alleles was detected in domesticated and wild emmer accessions (**Figure 5**). This trend of declining frequency of resistant alleles in commercial cultivars may reflect that the selection for other agronomically important traits may have reduced the frequency of FHB resistance in breeding programs. This may not be surprising, since many of the QTL we identified were associated with agro-morphological traits important to regional adaption (heading time, maturity time, plant height). For example, *QFhb-5A* was strongly associated with FHB resistance but, as we noted above, was associated with HD, AD, HT, and MAT (**Table 1**). Both HD and MAT must be optimized for regional adaptation and to maximize grain yield potential (Alahmad et al., 2020), and selection for these two traits may have unintendedly reduced the frequency of FHB resistance alleles. At the same time, in a study by Gaire et al. (2021), the accumulation of FHB-resistance loci showed reduced DON but resulted in lower yield potential, highlighting a trade-off between FHB resistance and grain yield.

The wild relatives of wheat are a valuable resource for FHB resistance in wheat, and various studies have identified moderate levels of resistance in the wild (Ruan et al., 2012), cultivated emmer wheat (Ruan et al., 2012; Ruan et al., 2020), and Persian wheat (*T. carthlicum;* Somers et al., 2006). In our study, the frequency of resistant haplotypes at the six prominent QTL (**Supplementary Table S6**) was higher in the wild and domesticated emmer accessions from our panel (**Supplementary Table S1**), supporting their use in breeding. For example, we included several elite progenies of lines from *T. carthlicum* cv. “Blackbird” (Somers et al., 2006) and *T. turgidum* ssp. *dicoccum* TG3487 (Ruan et al., 2012; Ruan et al., 2020). Their progeny (D04X_84_030, D04X_84_033, and D04X_84_104), P48_19, A1200J_101, and A1200K_209, P49_7, and R11_27_1, carried the favorable alleles for the five FHB resistance QTL. Thus, exotic and wild germplasm is an important source of FHB resistance, and we are currently pyramiding the QTL identified from these native sources of resistance. Of course, we are mindful that linkage drag with other important durum traits is a reality when using these materials—as we observed for PRO and YP—but in our experience, these negative associations can be overcome by using a combination of marker-assisted selection, genomic selection, and coselection for FHB-related traits and agronomic performance (Haile et al., 2019).

## 5 Conclusions

Identifying and utilizing novel QTL and genes for resistance is a continuous and regular challenge in plant breeding to deal with the threats to crop production caused by diseases. Genome-wide association studies are one of the strategies to detect QTL associated with resistance. In this study, by applying ML-GWAS models to a panel of 265 durum wheat cultivars, breeding lines, and experimental populations, we provided comprehensive insight into the molecular genetic basis of FHB resistance and correlated agro-morphological and quality traits. SNPs associated with FHB resistance were identified across the 14 chromosomes. Among the major QTL identified in this study, six QTL regions on chromosomes 2A, 2B, 3B, 5A, and 6B were the most consistent across traits and environments and are recommended for marker-assisted gene stacking. Although most of them are identified in known regions, *QFhb-3B.2* associated with FHB SEV, INC, and DON could enhance our understanding and provide new resources for FHB resistance breeding. Stacking the QTL identified in this study and using the lines carrying the resistance alleles will facilitate further genetic improvement of FHB resistance and reduce DON accumulation in durum wheat. However, it is vital to integrate trait associations into breeding decisions, particularly when using QTL such as *QFhb.4B.1* and *QFhb.5A*, which colocalized with multiple agro-morphological traits.

Lastly, based on the results of our and previous studies (Prat et al., 2017; Ruan et al., 2020), *Fhb1* is effective in diverse durum backgrounds and in combination with other resistance QTL. Therefore, we suggest that pyramiding *Fhb1* with resistance QTL derived from exotic germplasm (*T. turgidum* ssp*. dicoccoides* and *T. turgidum* ssp*. dicoccum*) could improve FHB resistance in durum wheat. Finally, we have developed robust KASP markers for the six prominent QTL associated with FHB resistance and quantified their haplotypes in the Global Durum Panel. These markers, together with their assessment of the GDP, will be a useful resource to support marker-assisted breeding and/or as main effect markers in genomic selection programs.

## Data availability statement

The datasets presented in this study can be found in online repositories. The names of the repository/repositories and accession number(s) can be found in the article/**Supplementary Material**.

## Author contributions

JH and CJP designed the experiments. JH, M-AH, VK, KW, and LW conducted experiments and collected data under the supervision of CP. JH, DS, AN, KW, and HC performed data analysis. JH prepared the manuscript with input from DS and AN. CP, VK, BS, HB, HK, DS, M-AH, LW, and YR reviewed and edited the manuscript. All authors contributed to the article and approved the submitted version.
