# Supplementary material for: Multi-locus genome-wide association studies reveal the genetic architecture of Fusarium head blight resistance in durum wheat
Source: Front Plant Sci. 2023 Oct 12;14:1182548. doi: 10.3389/fpls.2023.1182548 (PMC10601657; doi:10.3389/fpls.2023.1182548)
Supplement: Supplementary file 1 [file DataSheet_1.docx]

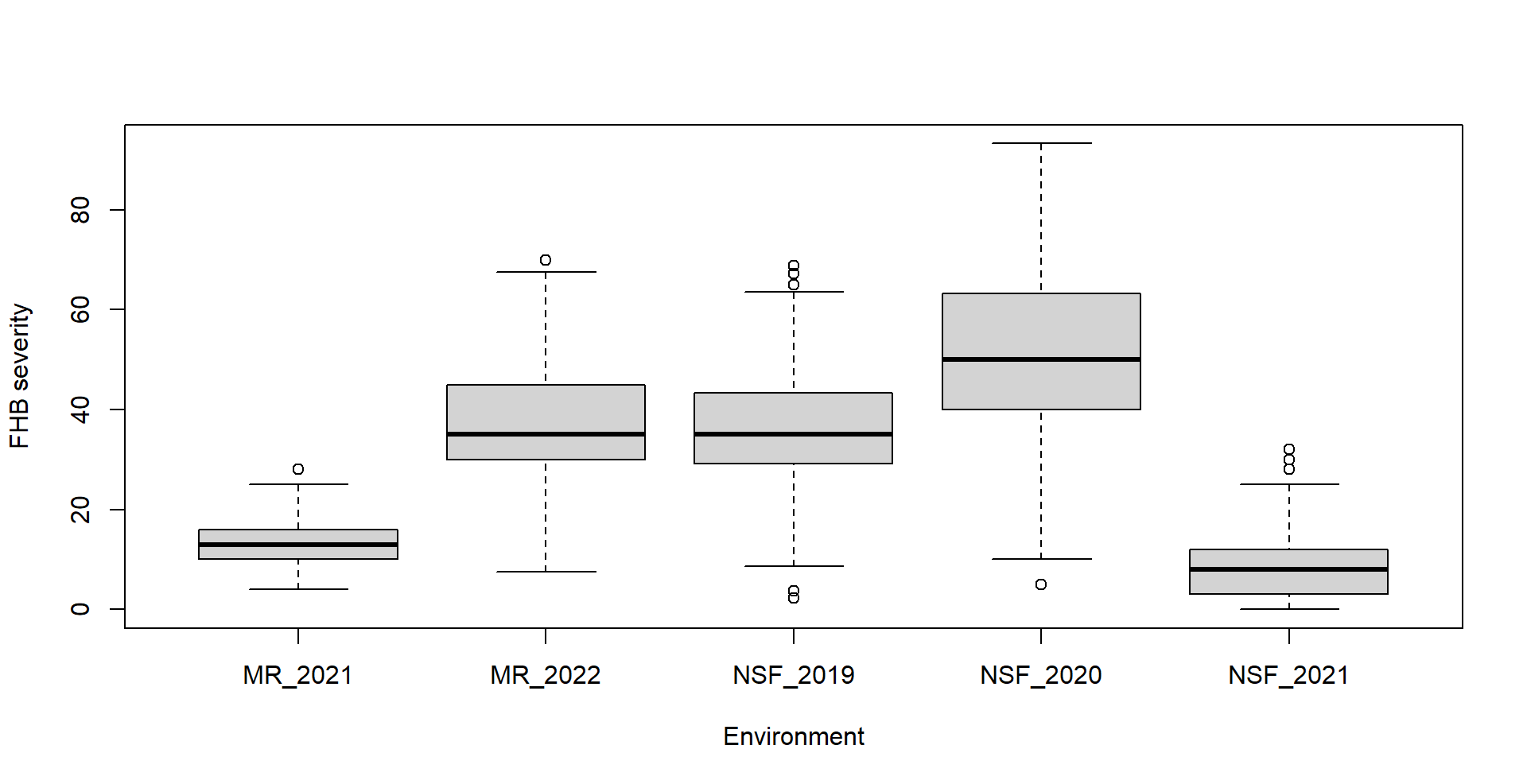


**Figure S1.** FHB severity (%) for the four consecutive years (2019, 2020, 2021 and 2022) at the two field nurseries, NSF (Saskatoon, SK) and MR (Morden, MB).


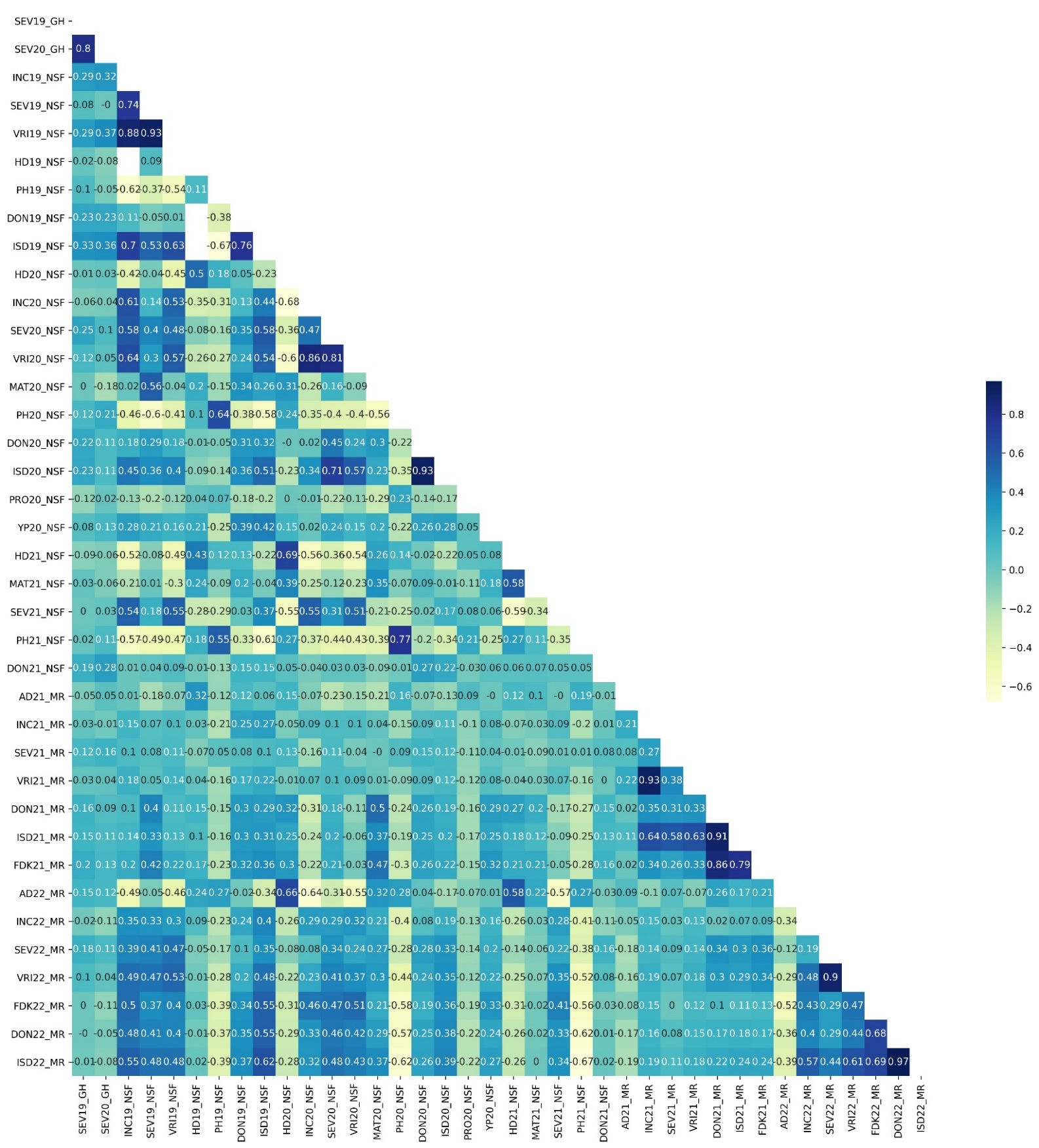


**Figure S2**. Correlation heatmap of FHB and other agro-morphological traits in the panel based on individual environment data.

**
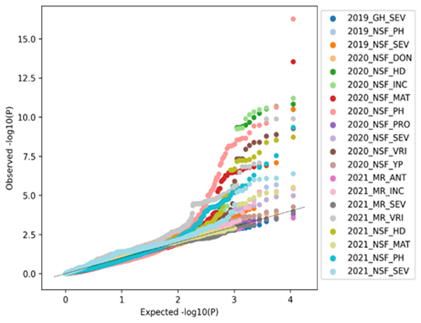
**

**Figure S3.** Quantile-quantile (Q-Q) plot showing the distribution of the data against the expected normal distribution for the tested traits across all environments using MLM of TASSEL.

**
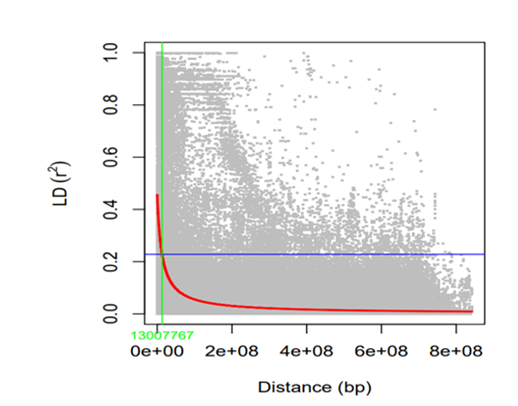
**

**
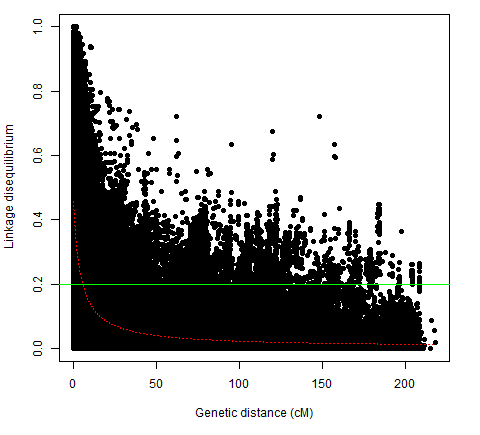
**

**Figure S4.** Decay of Linkage disequilibrium (r^2^) as a function of physical distance (13Mbp) (left) and genetic distance (5.8cM) (right) between pairs of loci the on all chromosomes (left). The blue dashed line represents the critical r^2^ and the red curve being the locally weighted scatterplot smoothing (LOESS) function.


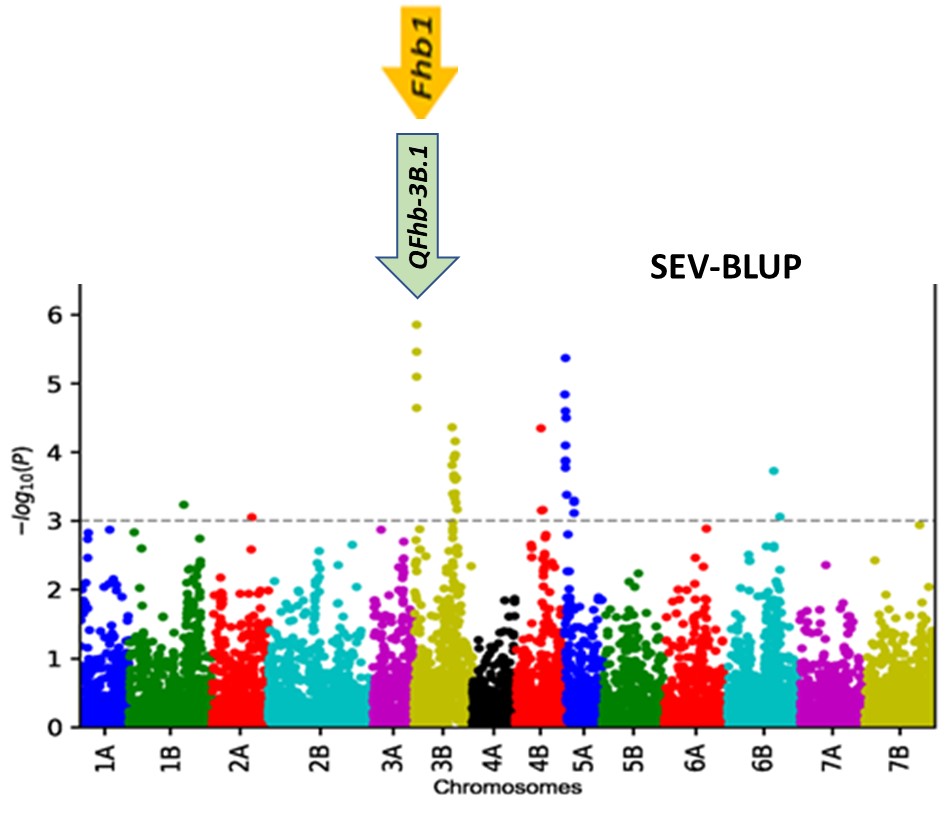


**Figure S5.** Manhattan plots reveal QTL *QFhb-3B.1* (near *Fhb1*) for BLUP value from five environments performed by the Tassel MLM model.


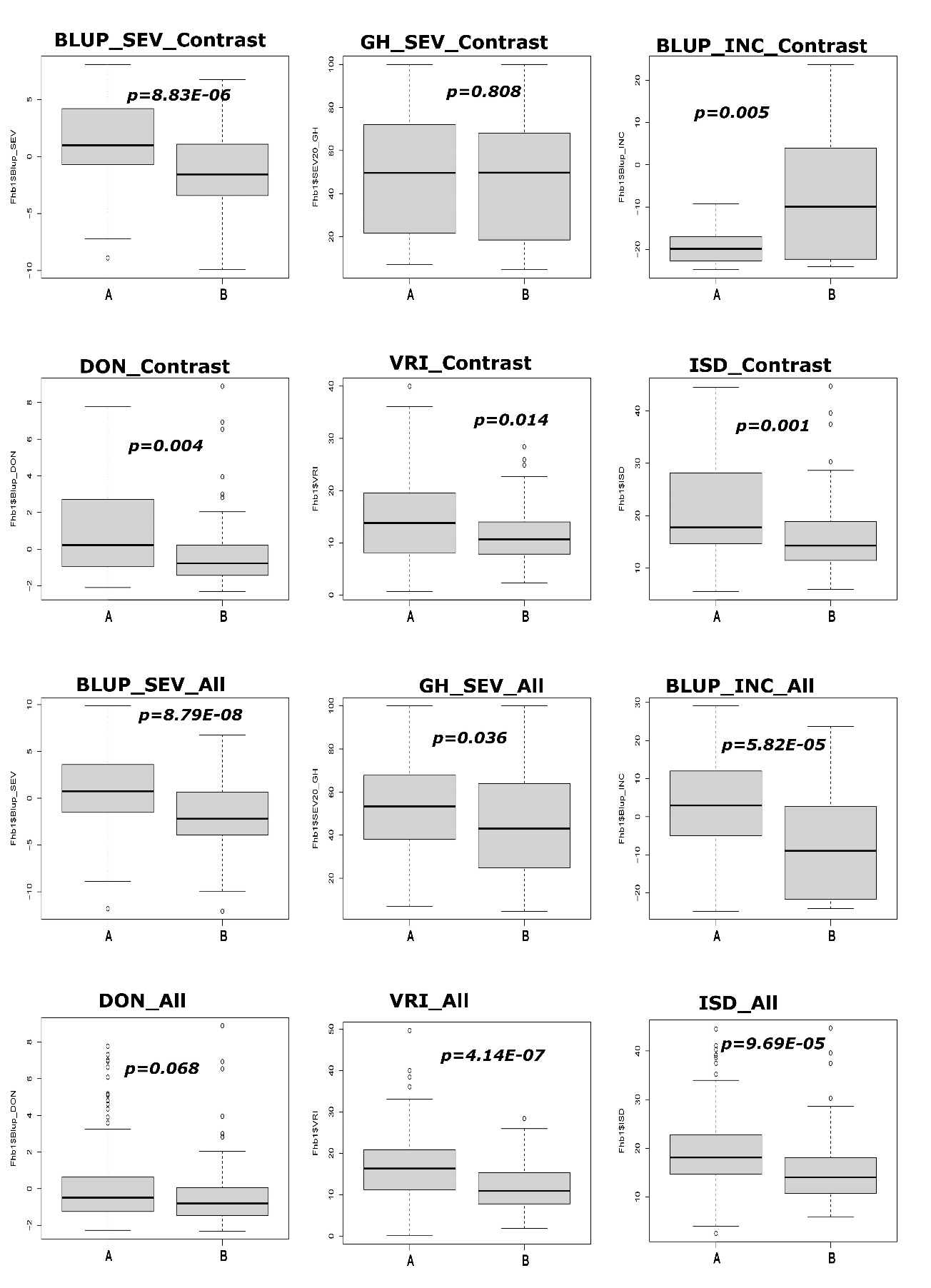


**Figure S6.** Boxplot distributions of lines according to their allele status at representative SNP *TA004185-0427* (3B) for SEV, INC, DON, VRI and ISD for known *Fhb1* carriers vs non-carriers (Top 2 rows) and all lines in the panel (bottom 2 rows). ‘**Contrast**’– indicates the comparison between known *Fhb1* carriers and non-carriers, whereas ‘**All**’ indicates all the lines in the panel used for this study.


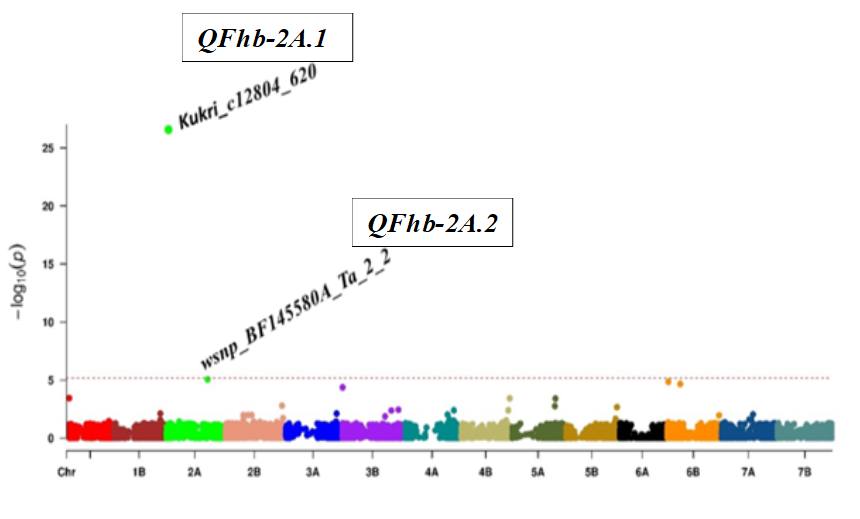


**Figure S7.** Manhattan plot of LD-block-based RTM GWAS for DON (GH). The x-axis indicates the number of LD blocks along the chromosomes. Each color in the Manhattan plot indicates a chromosome. The horizontal red line indicates the significant threshold p-value based on the Bonferroni multiple comparison correction methods at α=0.05 (0.05/n, where n is the number of LD-Blocks).


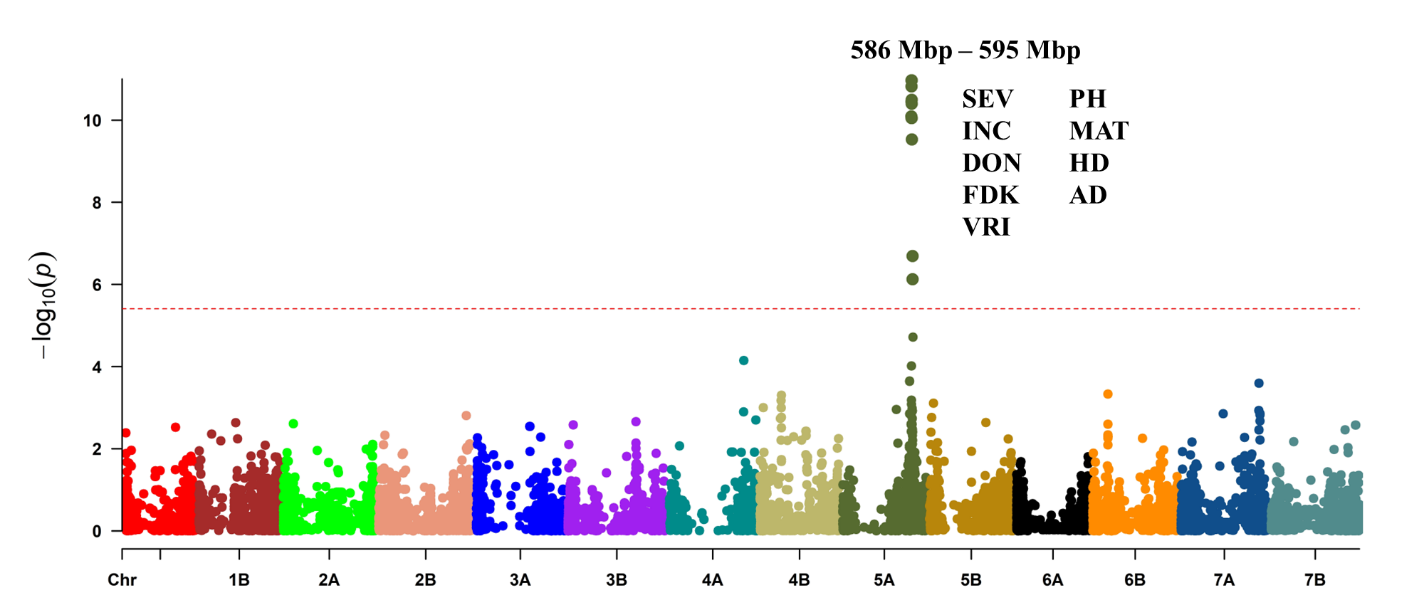


**Figure S8.** QTL region on chromosome 5A associated with multiple FHB resistance, and agro-morphological traits and detected by all the GWAS models used.


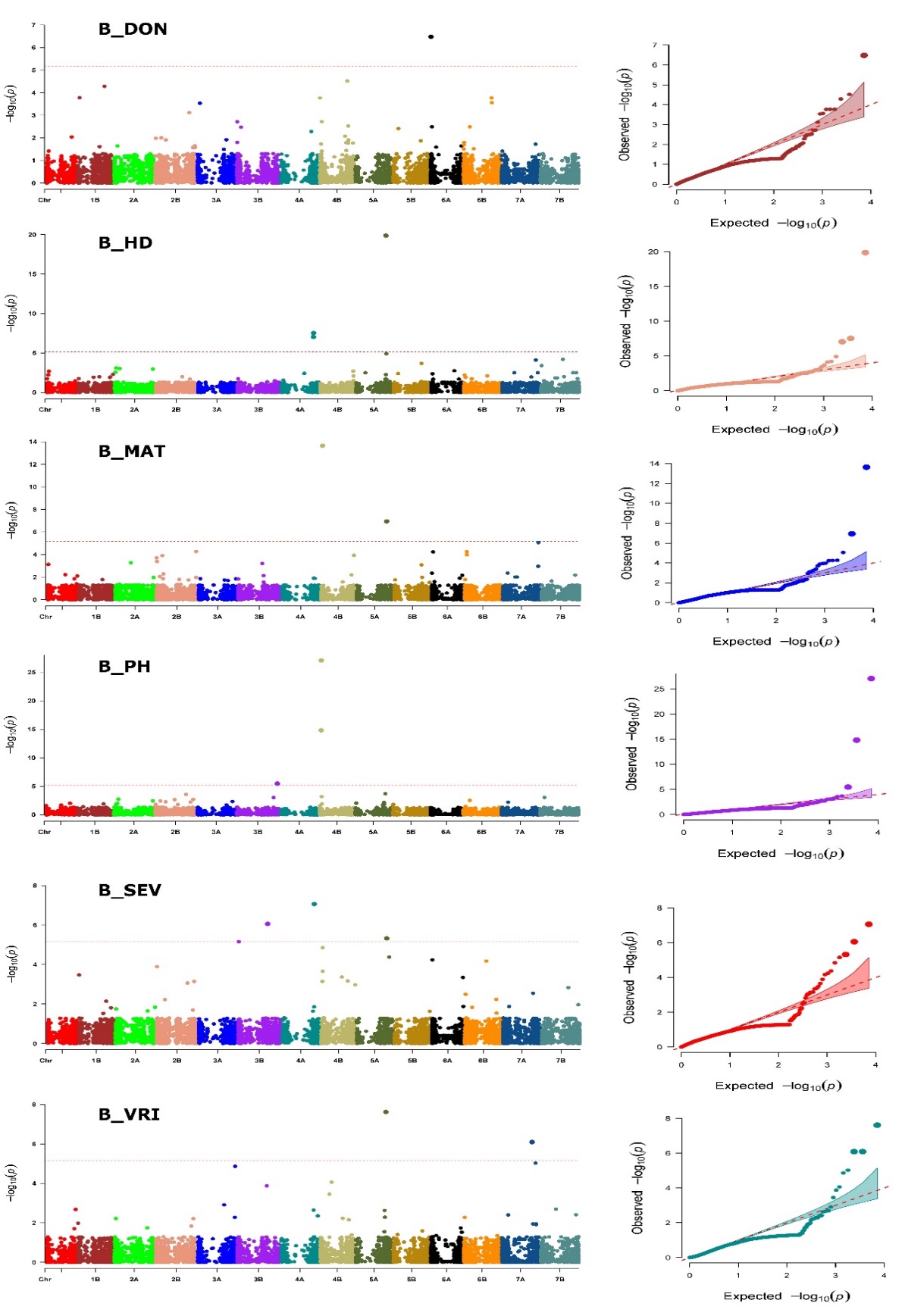


**Figure S9.** Manhattan plot for significance [–log10(p-values)] of the association of 13,787 SNPs based on RTM LdBlock-based method located on 14 chromosomes with BLUP values. Associations for deoxynivalenol (DON), heading date (HD), maturity date (MAT), plant height (PH), FHB severity (SEV) and FHB variety rating index (VRI) were plotted from top to bottom, respectively. A multi-track Q-Q plot for each case is presented at the right corner of the respective Manhattan plot. The threshold value at –log10(p) ≥ 5 is indicated as a horizontal line. B – BLUP value


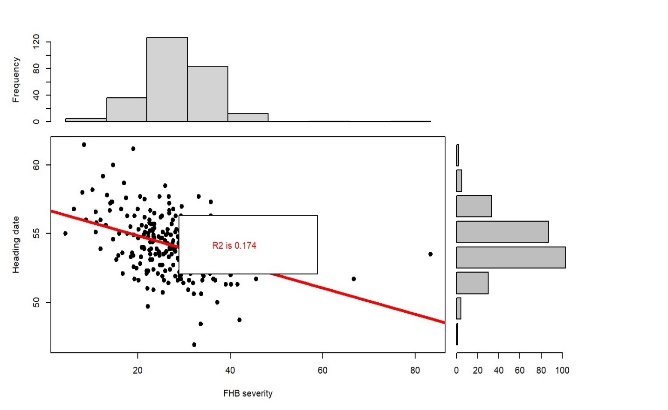

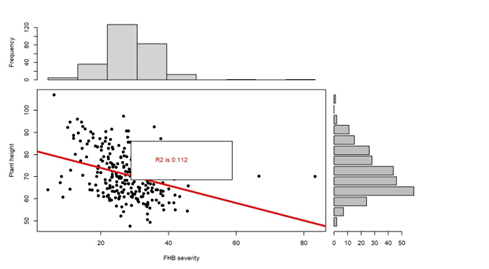


**Figure S10.** Relationship between heading date (left) and plant height (right) with FHB severity in the panel


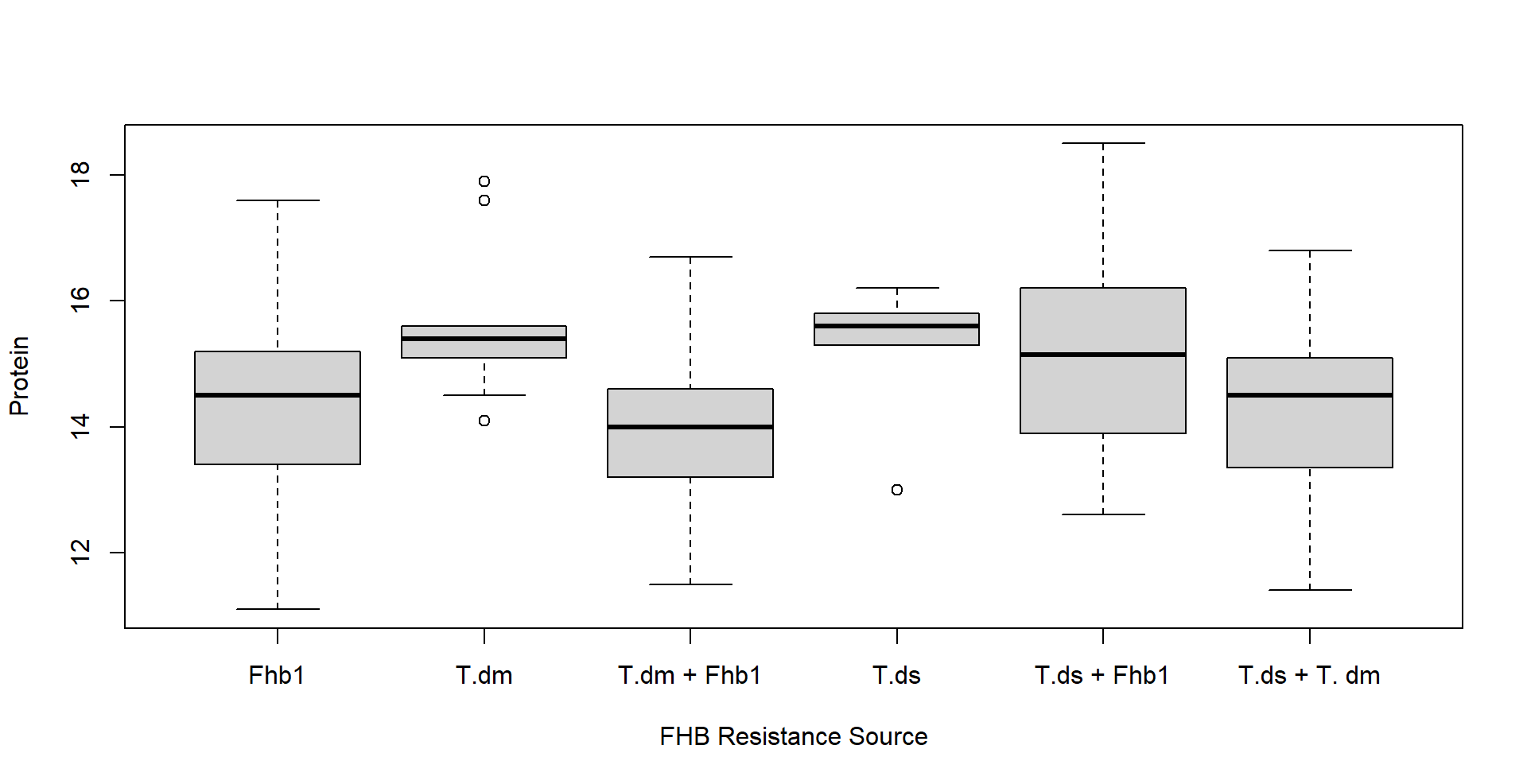


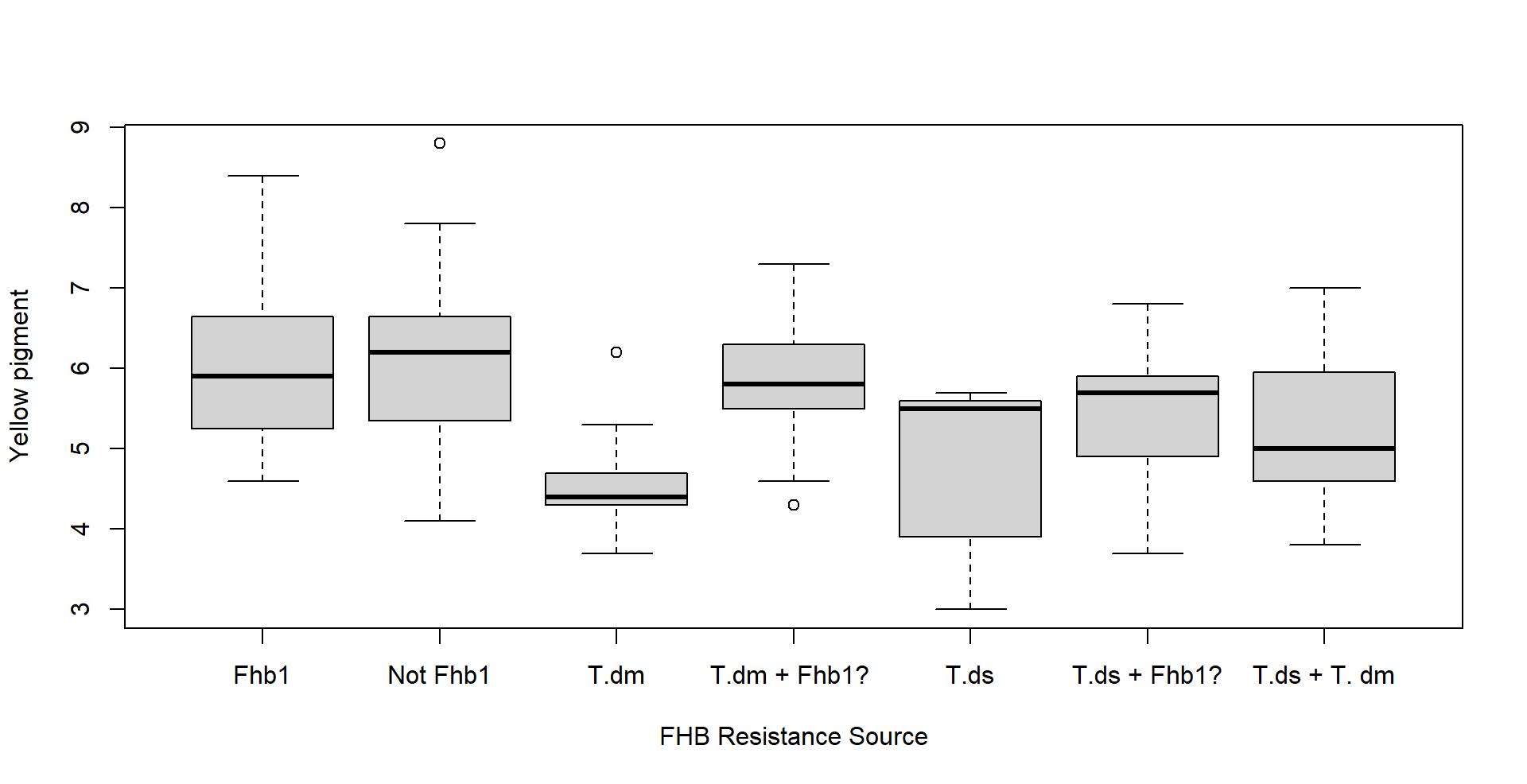


**Figure S11.** Protein content (top) and yellow pigment (bottom) analyzed from the DON sample collected from the 2020 North Seed Farm (NSF_2020) and plotted based on different FHB-resistant backgrounds. T.dm – *T. turgidum* ssp. *dicoccum*; T.ds - and *T. turgidum* ssp. *dicoccoides*


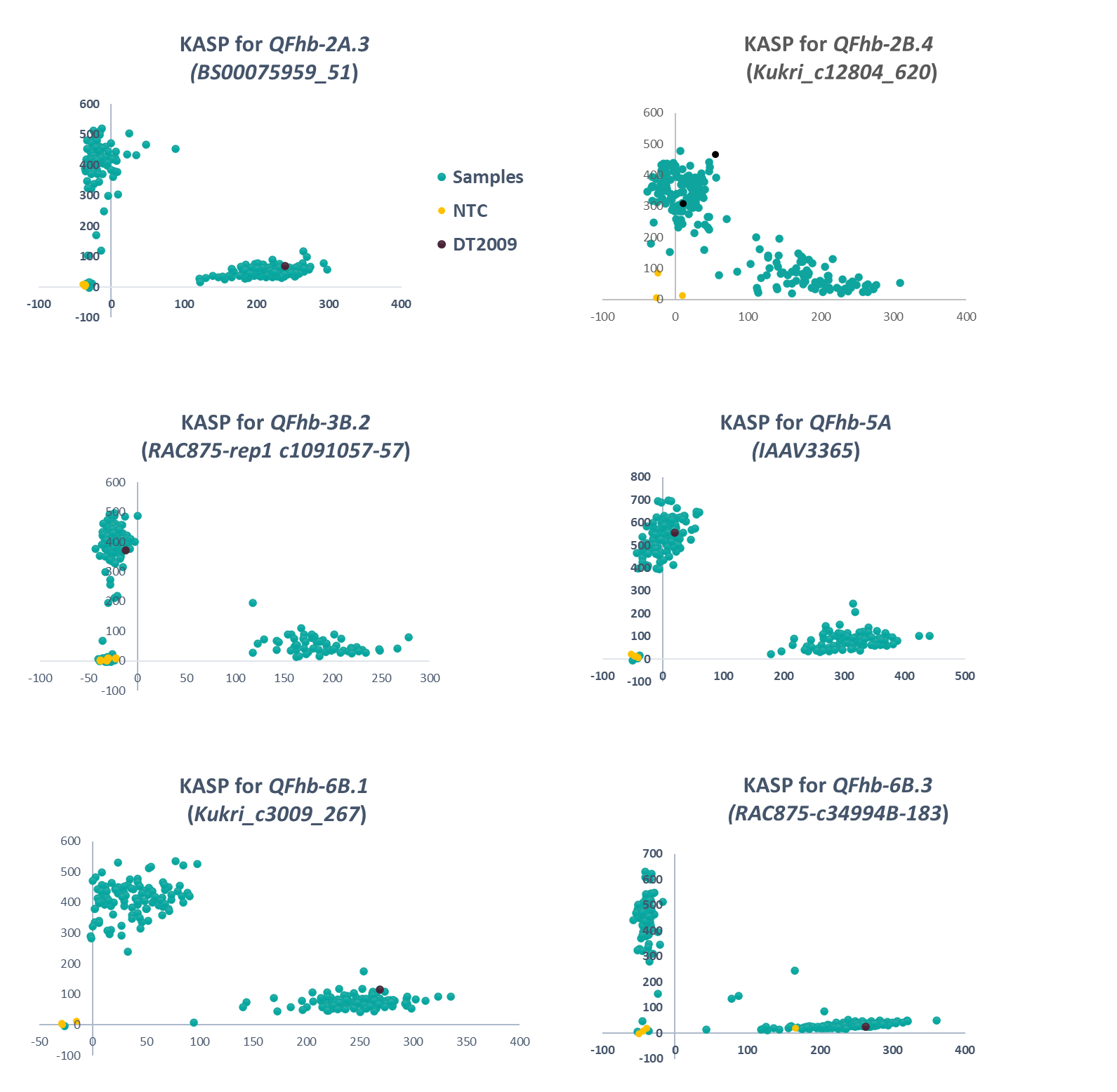


**Figure S12.** KASP markers for QTL *QFhb-2A.3*, *QFhb-2B.4,* *QFhb-3B.2,* *QFhb-5A*, *QFhb-6B.1* and *QFhb-6B.3* validated on Durum Global Diversity Paneld (GDP). DT2009 (AAC Schrader) is an FHB intermediately resistant cultivar.
