# Supplementary material for: Multi-locus genome-wide association studies reveal the genetic architecture of Fusarium head blight resistance in durum wheat
Source: Front Plant Sci. 2023 Oct 12;14:1182548. doi: 10.3389/fpls.2023.1182548 (PMC10601657; doi:10.3389/fpls.2023.1182548)
Supplement: Supplementary file 3 [file Table_2.docx]

**Table S2.** Phenotypic performance of the 265 lines under field and GH conditions.

| **Trait** | **Environment** | **Mean** | **Range**  **(Min and Max)** | **SD** | **Across environment Broad sense heritability (H^2^, %)** |
| --- | --- | --- | --- | --- | --- |
| **FHB severity** | 2019_NSF | 36.17 | 2-69 | 12.23 | 0.61 |
|  | 2020_NSF | 50.34 | 5-93 | 16.87 |  |
|  | 2021_NSF * | 8.55 | 2-32 | 6.24 |  |
|  | 2021_MR | 13.13 | 4-28 | 4.39 |  |
|  | 2022_MR | 37.94 | 8-70 | 10.74 |  |
|  | 2019_GH | 51.20 | 5-100 | 22.48 | 0.72 |
|  | 2020_GH | 50.6 | 5-100 | 23.1 |  |
|  |  |  |  |  |  |
| **FHB incidence** | 2019_NSF | 54.69 | 7-87 | 13.91 | 0.38 |
|  | 2020_NSF | 41.78 | 7-77 | 14.74 |  |
|  | 2021_MR | 5.11 | 1-32 | 5.29 |  |
|  | 2022_MR | 84.61 | 20-98 | 13.27 |  |
|  |  |  |  |  |  |
| **FHB index (VRI)** | 2019_NSF | 18.10 | 0-46 | 8.04 |  |
|  | 2020_NSF | 22.20 | 0-65 | 12.22 |  |
|  | 2021_MR | 0.84 | 0-9 | 1.18 |  |
|  | 2022_MR | 32.5 | 4-67 | 11.4 |  |
|  |  |  |  |  |  |
| **DON** | 2019_NSF | 10.18 | 0.740-37.630 | 7.66 | 0.86 |
|  | 2020_NSF | 15.04 | 0.000-97.600 | 19.16 |  |
|  | 2021_NSF | 2.59 | 0.000-37.544 | 3.99 |  |
|  | 2021_MR | 4.96 | 0.400-19.800 | 3.92 |  |
|  | 2020_GH | 33.44 | 0.377-228.800 | 38.80 |  |
|  | 2022_MR | 47.82 | 5.594-94.224 | 10.02 |  |
|  |  |  |  |  |  |
| **ISD** | 2019_NSF | 23.48 | 3-40 | 6.28 |  |
|  | 2020_NSF | 27.44 | 3-79 | 14.08 |  |
|  | 2021_MR | 6.60 | 1-18 | 3.30 |  |
|  | 2022_MR | 53.67 | 15-85 | 13.40 |  |
|  |  |  |  |  |  |
| **FDKs** | 2021_MR | 3.21 | 0-20 | 2.90 | 0.65 |
|  | 2022_MR | 17.20 | 0.2-41 | 9.48 |  |
|  |  |  |  |  |  |
| **HD** | 2019_NSF | 38.53 | 34-41 | 1.32 | 0.84 |
|  | 2020_NSF | 55.49 | 50-60 | 1.76 |  |
|  | 2021_NSF | 55.20 | 48-65 | 2.00 |  |
|  |  |  |  |  |  |
| **AD** | 2021_MR | 63.68 | 54-74 | 3.48 | 0.59 |
|  | 2022_MR | 58.78 | 51-69 | 4.54 |  |
|  |  |  |  |  |  |
| **MAT** | 2020_NSF | 90.38 | 85-100 | 3.17 | 0.42 |
|  | 2021_NSF | 89.13 | 84-97 | 1.87 |  |
|  |  |  |  |  |  |
| **PH** | 2019_NSF | 76.70 | 52-113 | 11.76 | 0.93 |
|  | 2020_NSF | 83.22 | 52-116 | 13.58 |  |
|  | 2021_NSF | 56.35 | 37-94 | 8.61 |  |

**Where:** SD -standard deviation, FHB – *Fusarium* head blight, DON – deoxynivalenol, ISD –INC-SEV-DON index, FDKs – F*usarium* damaged Kernels, HD – heading date, AD – anthesis date, MAT – maturity date, PH – plant height, NSF-North Seed Farm (FHB screening nursery at Saskatoon, SK), MR- Morden FHB screening nursery, MB; H^2^- trait's broad sense heritability.
