# Supplementary material for: Multi-locus genome-wide association studies reveal the genetic architecture of Fusarium head blight resistance in durum wheat
Source: Front Plant Sci. 2023 Oct 12;14:1182548. doi: 10.3389/fpls.2023.1182548 (PMC10601657; doi:10.3389/fpls.2023.1182548)
Supplement: Supplementary file 5 [file Table_4.docx]

**Table S4.** QTNs detected by two or more GWAS models and traits/environments for FHB resistance traits

| QTL | SNPs | Chr | Marker position (Mbp) | **Trait** (Environment) | LOD score | R^2^ (%) | -log10(P) | MAF | Method |
| --- | --- | --- | --- | --- | --- | --- | --- | --- | --- |
| *QFhb-1A.1* | *Ra_c4159_2716* | 1A | 490.5 | **DON** (GH) **INC** (19NSF) **SEV** (19GH) | 3.6 - 3.8 | 9.1 **- 35.7** | 4.37 - 4.58 | 0.3 | 1, 6 |
| *QFhb-1A.2* | *TA003794-1077, Kukri-Ra_c5683-2584* | 1A | 562.9 | **INC** (21MR, 20NSF) | 3.4 - 3.6 | 5.0 - 6.2 | 4.15 - 4.33 | 0.33 | 1, 6 |
| *QFhb-1B* | *BS00029013_51* | 1B | 109.3 | **INC** (BLUP, 20NSF), **VRI** | 3.7 - 7.4 | 7.0 - **26.4** | 4.43 - 8.28 | 0.27 | 1, 6 |
| *QFhb-2A.1* | *BS00098033_51* | 2A | 778.7 | DON (GH) | 3.4 | 5.2 | 4.11 | 0.23 | 1, R |
| *QFhb-2A.2* | *wsnp_BF145580A_Ta_2_2* | 2A | 568.4 | I**NC** (19NSF) **SEV** (19NSF) | 3.8 - 5.8 | 6.7 - 10.3 | 4.49 - 6.60 | 0.49 | 1, 4, 5, 6 |
| *QFhb-2A.3* | *BS00000209_51* | 2A | 746.8 | **DON** (BLUP, 20NSF) **SEV** (BLUP, 19GH, 20GH, 19NSF, 21NSF, 21MR) | 3.3 - 6.1 | **23.2 - 24.0** | 4.05 - 6.95 | 0.32 | 3, 5, 6 |
| *QFhb-2B.1****** | *BobWhite_c12144_216* | 2B | 10.8 | **SEV** (19NSF) **PH** | 4.3 - 5.5 | 10.7 | 5.09 - 6.29 | 0.34 | 3, 4 |
| *QFhb-2B.2* | *Ex_c55735_1012* | 2B | 605.0 | **SEV** (21MR) | 3.5 - 4.6 | **20.1 - 21.0** | 4.24 - 5.41 | 0.41 | 4, 5, 6 |
| *QFhb-2B.3* | *Excalibur_c39451_68,*  *Ku_c3479_1035* | 2B | 683.2 | **DON** (19NSF) **SEV** (BLUP, 20GH, 20NSF, 21NSF) | 4.3 - 6.4 | 12.1 **- 16.9** | 5.02 - 7.28 | 0.47 | 1, 4, 5, 6 |
| *QFhb-2B.4* | *Kukri_c12804_620* | 2B | 114 | **DON (GH) SEV (GH) INC (21MR)** | 3.3 - 4.8 | 5.3 – 15.1 | 4.05 - 5.54 | 0.48 | 1, 4, 5, 6, R |
| ***QFhb-3A.1*** | *RAC875_c4954_943 wsnp_Ex_c23633_32868822* | 3A | 10.0–13.0 | **SEV** (20NSF), **ISD PRO** | 3.3 - 6.2 | 5.6 - 7.8 | 4.04 - 7.02 | 0.46 | 3, 5, 6 |
| *QFhb-3A.2* | *RAC875_c10710_87* | 3A | 716.1 | **SEV** (GH) | 3.7 - 3.9 | 7.9 - 11.8 | 4.48 - 4.69 | 0.44 | 5, 6 |
| *QFhb-3B.1* | *TA004185-0427*  *RAC875_c5966_1854* | 3B | 3.2 -9.9 | **SEV** (BLUP)  **INC** (22MR) **DON** (BLUP), **FDK**, **ISD** | 3.6 - 5.0 | 7.2 - **20.1** | 3.87- 5.73 | 0.46 | 2, 4,3, 5, T |
| *QFhb-3B.2* | *RAC875_rep_c109105_57, Excalibur_c15332_453, RAC875_rep_c115516_134,* *Excalibur_c62826_254* | 3B | 578.2-578.8 | **SEV** (BLUP, 20NSF) **DON** (21MR, 20NSF, BLUP) **INC** (BLUP), **VRI** | 3.2 - 11.9 | 10.5 - **36.9** | 4.64 - 12.84 | 0.37 | 1, 3, 4, 5, 6, R, T |
| *QFhb-3B.3* | *BobWhite_c6462_373* | 3B | 793.1 | **SEV** (20GH, BLUP, 19GH) | 3.4 - 5.6 | 13.1 **- 24.9** | 4.08 - 6.41 | 0.36 | 1, 3, 4, 5 |
| *QFhb-4A.1* | *Kukri_c53787_620* | 4A | 21.2 | **SEV** (21NSF) | 4.8 - 6.1 | 10.5 **- 27.6** | 5.6 - 7.0 | 0.41 | 1, 3, 4, 5, 6, T |
| *QFhb-4B.1******* | *wsnp_BF482960B_Ta_1_4, RAC875_c27536_611, BS00021984_51, Ex_c101685_711* | 4B | 29.0-35.5 | **SEV** (19NSF) **INC** (22MR) **DON** (21MR, 22MR),  **FDK** (21MR) **PH, MAT** | 3.8- 20.1 | 5.3 **– 52.3** | 4.52 - 21.23 | 0.29 | 1, 2, 3, 4, 5, 6, R, T |
| *QFhb-4B.2* | *Tdurum_contig12177_1367* | 4B | 88.8 | **INC** (19NSF) | 4.9 - 6.9 | **17.3 - 27.5** | 5.68 - 7.80 | 0.42 | 5, 6 |
| *QFhb-4B.3* | *Tdurum_contig14562_607* | 4B | 181.7 | **INC** (BLUP) | 3.9 - 5.2 | 10.1 - 11.3 | 4.64 - 6.03 | 0.38 | 3, 4, 6 |
| *QFhb-4B.4* | *Ra_c41921_951* | 4B | 558.1 | **DON** (BLUP, 20NSF), **ISD** | 3.7 - 6.1 | 5.5 - 8.6 | 4.40 - 6.89 | 0.47 | 3, 4, 5, 6, R, T |
| *QFhb-4B.5* | *Kukri_c26905_392* | 4B | 657.8 | **SEV** (21NSF) **DON** (GH) | 3.3 - 6.2 | 6.6 - 13.3 | 4.01 7.01 | 0.45 | 3, 4, 5, 6 |
| *QFhb-5A******** | *IAAV3365, BS00075959_51, wsnp_AJ612027A_Ta_2_5, BobWhite_c21949_150, wsnp_BF293620A_Ta_2_1, Kukri_c33022_198, RFL_Contig316_572, Excalibur_c23354_306* | 5A | 586.6- 595.4 | **INC** (19NSF, 20NSF, BLUP) **SEV** (20NSF, 21NSF, BLUP) **DON (**21MR, 22MR)  **FDK** (21MR, 22MR)  **VRI HD, AD, MAT, HT** | 3.3 - 21.9 | 6.0 – **44.5** | 3.74 - 23.04 | 0.40 | 1, 2, 3, 4, 5 6, R, T |
| *QFhb-5B.1* | *wsnp_Ra_c24619_34168104* | 5B | 508.8 | **DON** (19NSF) **SEV** (BLUP, 19GH, 20GH) | 5.1 - 7.1 | **36.5 - 53.4** | 5.9 - 7.9 | 0.23 | 1, 4, 5, 6 |
| *QFhb-5B.2* | *Ra_c2216_1442* | 5B | 591.1 | **SEV** (20GH), **FDK** | 4.2 - 6.0 | 6.2 - 13.0 | 4.99 - 6.83 | 0.31 | 1, 4, 6 |
| *QFhb-6A******* | *Ra_c29107_289* | 6A | 18.5 | **DON** (GH) **INC** (BLUP) **MAT** | 4.1 - 4.4 | **15.0 - 20.2** | 4.81- 5.14 | 0.42 | 4, 5 |
| *QFhb-6B.1* | *Excalibur_c30648_924, RAC875_c12259_1892, RAC875_c20634_100, Kukri_c3009_267, Excalibur_c30648_868* | 6B | 11.5-18.5 | **SEV** (19GH, 20GH, BLUP, 20NSF) **DON** (19NSF, BLUP, 20NSF) **ISD** | 3.9 - 9.1 | 5.7 - **26.6** | 4.68 - 9.98 | 0.30 | 1, 2, 3, 4, 5 6, T |
| *QFhb-6B.2* | *Kukri_c21995_1652* | 6B | 47.0 | **SEV** (20GH) **DON** (GH) | 4.0 - 4.7 | 9.8 **- 40.0** | 4.77 - 5.43 | 0.32 | 3, 4, T |
| *QFhb-6B.3* | *Tdurum_contig45714_427, RAC875_c34994_183, Kukri_c75566_265* | 6B | 123.8-128.7 | **INC** (BLUP, 21MR) **SEV** (BLUP, 21NSF, 21MR) **DON** (GH, 19NSF, 20NSF, BLUP) | 3.5 - 6.7 | 5.2 - 13.8 | 4.18 - 7.55 | 0.41 | 2, 3, 4, 5, 6 |
| *QFhb-7A******* | *Tdurum_contig69067_405* | 7A | 662.0- 671.0 | **DON** (GH), **VRI HD** | 4.8 - 5.3 | 5.4 - **37.0** | 5.54 - 6.12 | 0.11 | 5, 6, T |
| *QFhb-7B.1******* | *Kukri_c51101_351* | 7B | 630.1 | **DON** (GH) **SEV** (BLUP, 20GH, 21MR) **HD, MAT** | 3.4 - 6.0 | 11.2 **- 24.1** | 4.14 - 6.79 | 0.45 | 4, 5, 6 |
| ***QFhb-7B.2*** | *Excalibur_c49736_1197,*  *IAAV3713* | 7B | 706.9 -728.7 | **DON** (GH) **SEV** (19GH, 20GH, BLUP)  **YP** | 4.1 - 6.8 | 14.8 **- 19.4** | 4.90- 7.70 | 0.48 | 4, 5 |

**Models:** 1= MrMLM, 2=FASTmrEMMA, 3=pKWmEB, 4=ISIS EM-BLASSO, 5=FASTmrMLM , 6= pLARmEB, R=RTM and T=Tassel

* Co-localized with PH, ** Colocalized with MAT and/or AD/HD, *** Colocalized with MAT, PH and AD/HD

QTL in bold are associated with quality traits; Marker positions are based on Chinese Spring reference genome v1

R^2^ in bold – contributed >15% to the phenotypic variation

**Traits: SEV** – FHB severity, **INC** – FHB incidence, **DON** – Deoxynivalenol, **FDK** – Fusarium damaged kernel. **VRI** – Visual rating index (SEV * INC/100), **ISD** = INC- SEV-DON index (0.2*SEV+0.2*INC+0.6*DON), **HD** – days to heading, **AD** – days to anthesis, **MAT** – days to maturity, **PH** – plant height. **YP** – yellow pigment, **PRO** – Grain protein content

**Location and year**: NSF – North Sed Farm, Saskatoon, SK; MR – Morden, MB; GH - Greenhouse; 19 – 2019, 20 – 2020, 21 – 2021, 22 – 2022
