# Supplementary material for: Multi-locus genome-wide association studies reveal the genetic architecture of Fusarium head blight resistance in durum wheat
Source: Front Plant Sci. 2023 Oct 12;14:1182548. doi: 10.3389/fpls.2023.1182548 (PMC10601657; doi:10.3389/fpls.2023.1182548)
Supplement: Supplementary file 6 [file Table_5.docx]

| QTL Name | *SNP* | Chr | Marker position (Mbp) | **Trait** (Environment) | LOD score | R^2^ (%) | -log10(P) | MAF | Model |
| --- | --- | --- | --- | --- | --- | --- | --- | --- | --- |
| *Q1B1.1* | *BS00095286_51* | 1B | 8.9 | **PH** (21NSF) **MAT** (20NSF, BLUP) | 4.9 - 6.7 | 5.7 - 11.0 | 11.07 - 12.56 | 0.23 | 1, 5, T |
| *Q1B1.2* | *wsnp_BM140362B_Ta_1_1* | 1B | 553.6 | **AD** (21MR) | 3.6 - 4.6 | **18.5 - 23.0** | 4.30- 5.40 | 0.33 | 4, 5 |
| *Q2B1.1* | *RAC875_rep_c71112_400* | 2B | 5.0 | **MAT** (20NSF) | 5.4 - 6.5 | 6.2 -7.4 | 9.90 - 17.62 | 0.14 | 3, 4 |
| *Q2B1.2* | *wsnp_Ra_c407_862316* | 2B | **53.5** | **PH** (21NSF) | 4.8 - 5.3 | 5.6 - 6.1 | 6.23 - 12.09 | 0.14 | 4, 6 |
| *Q3A* | *BS+B22:K2200084348_51* | 3A | **9.9** | **PRO** (NSF20) | 3.6 - 4.6 | 5.3 - 7.5 | 4.37 - 5.37 | 0.08 | 3, 4, 5, T |
| *Q4A* | *JG_c1844_303* | 4A | 589.6 | **PH** (19NSF, 20NSF, 21NSF, BLUP) **MAT** (20NSF, BLUP) | 4.7 - 6.5 | 5.5 - 13.0 | 9.45 - 31.91 | 0.21 | 5, 6, T |
| *Q4B.1* | *BS00021984_51, Ex_c101685_711, Tdurum_contig63670_287, Tdurum_contig93615_540, wsnp_BE442666B_Ta_2_2, wsnp_BF482960B_Ta_1_4* | 4B | **22.6-36.6** | **PH** (19NSF, 20NSF, 21NSF, BLUP) **MAT** (20NSF, BLUP) | 3.8 - 43.0 | 5.4- **60.3** | 3.94 - 62.11 | 0.19 | 1, 2, 3, 4, 5, 6, T, R |
| *Q4B.2* | *Tdurum_contig52805_183* | 4B | 404.5 | **HD** (21NSF, BLUP) | 3.3 - 4.1 | 5.4 - 9.3 | 3.97 - 4.89 | 0.35 | 3, 4 |
| *Q5A.1* | *BobWhite_c21949_150, IAAV3365, Kukri_c33022_198, Tdurum_contig52695_388, wsnp_Ex_c18754_27630557, wsnp_Ex_c54193_57155537* | 5A | **578.4-595.2** | **HD** (20NSF, 21NSF, BLUP)  **MAT** (21NSF, BLUP) **AD** (22MR) **HT** (21NSF) | 3.2 - 25.0 | 5.9 **-44.5** | 3.95 - 28.49 | 0.37 | 1, 2, 3, 4, 5, 6, T, R |
| *Q5A.2* | *TA001523-1009* | 5A | 659.7 | **MAT** (21NSF) **AD** (21MR) | 3.5 - 6.4 | 5.6 - 12.9 | 4.22 - 14.47 | 0.46 | 4, 5, 6 |
| *Q5B* | *BS00022652_51* | 5B | 378.9 | **PRO** (NSF20) | 3.7 | 4.27 | 8.5 | 0.49 | 5, T |
| *Q6A.1* | *BS00110902_51, wsnp_Ex_rep_c67468_66069282, Excalibur_c25211_828* | 6A | **23.4-34.3** | **MAT** (21NSF, BLUP) **PRO** (NSF20) | 3.6 - 8.0 | 6.3 - **29.7** | 4.29 - 14.85 | 0.40 | 1, 3, 4, 5, T |
| *Q6A.2* | *Tdurum_contig13068_208, Excalibur_c44739_232* | 6A | 595.1-608.5 | **HD** (19NSF) **YP** (NSF20) | 3.8 - 7.3 | 6.2 **- 29.5** | 4.51- 8.19 | 0.48 | 4, 5, 6 |
| *Q7A* | *wsnp_Ku_c42539_50247597* | 7A | 670.8 | **HD** (20NSF, BLUP) | 4.7 -4.9 | 8.7 - 11.1 | 5.5 -5.6 | 0.11 | 4, 5, T |
| *Q7B.1* | *JD_c11426_334* | 7B | 138.5 | **YP** (NSF20) | 5.0 - 6.1 | **22.2 - 24.8** | 5.80 - 6.96 | 0.08 | 5, 3, 4 |
| *Q7B.2* | *Kukri_c50071_1084* | 7B | **632.5** | **HD** (21NSF) **MAT** (21NSF, BLUP) | 3.6 - 5.9 | 5.9 - 6.8 | 4.29 - 25.57 | 0.32 | 3 |
| *Q7B.3* | *Kukri_c64387_218* | 7B | 595.2 | **HD** (BLUP) | 4.5 - 5.1 | 8.0 **- 21.6** | 5.24 - 5.94 | 0.25 | 1, 5 |
| *Q7B.4* | *Excalibur_c49736_1197* | 7B | **706.9** | **YP** (NSF20) | 5.2 - 8.2 | 7.6 **- 22.9** | 6.01 - 9.05 | 0.41 | 3, 4, 5, 6, T |

**Table S5.** QTNs detected by two or more GWAS models for agro-morphological and quality traits only BLUP values

SNPs highlighted - intervals co-localized with FHB response QTL regions, other footnotes are similar with **Table S4**
