# Supplementary material for: Multi-locus genome-wide association studies reveal the genetic architecture of Fusarium head blight resistance in durum wheat
Source: Front Plant Sci. 2023 Oct 12;14:1182548. doi: 10.3389/fpls.2023.1182548 (PMC10601657; doi:10.3389/fpls.2023.1182548)
Supplement: Supplementary file 7 [file Table_6.docx]

**Table S6.** Putative QTL and mean values of DON (ppm) of the lines in the panel

| **QTL** | ***QFhb-1A.1*** | ***QFhb-2A.3*** | ***QFhb-6A*** | ***QFhb-6B.1*** | ***QFhb-7B.2*** | **Mean DON** |
| --- | --- | --- | --- | --- | --- | --- |
| Representative SNP | *Ra_c4159_2716* | *BS00000209_51* | *Ra_c29107_289* | *Kukri_c3009_267* | *IAAV3713* |  |
| Favorable Allele | A | A | B | A | B |  |
| Kyle | - | + | - | - | + | 6.369 |
| Pithless_1 | - | - | - | - | + | 0.967 |
| Svevo | - | + | U | + | - | 6.511 |
| TG3487 | + | + | + | + | + | 7.343 |
| D04X_84_030 | + | + | + | + | + | 0.462 |
| D04X_84_033 | + | + | + | + | + | 2.247 |
| D04X_84_088 | - | + | + | + | + | 2.245 |
| D04X_84_104 | + | + | + | + | + | 0.987 |
| AAC_Congress | - | - | + | U | + | 2.050 |
| CDC_Precision | - | - | + | + | + | 1.250 |
| AAC_Succeed | - | - | + | + | + | 5.366 |
| CDC_Vivid | - | + | U | + | + | 9.313 |
| CDC_Credence | - | + | + | - | + | 2.085 |
| CDC_Dynamic | - | - | + | + | + | 2.141 |
| AAC_Stronghold | - | - | + | + | + | 9.793 |
| DT1003 | + | + | - | - | + | 2.207 |
| DT1004 | - | - | + | + | + | 1.834 |
| W9262_260D3 | + | - | - | + | + | 0.624 |
| Kofa | - | - | + | - | + | 24.213 |
| A0022_C679 | - | - | - | + | + | 12.508 |
| A0022_E872 | + | + | + | + | + | 1.456 |
| DT591 | - | + | + | - | + | 8.995 |
| CDC_Fortitude | - | - | + | - | + | 3.954 |
| AAC_Cabri | - | + | + | + | + | 6.694 |
| CDC_Carbide | - | - | + | + | + | 3.115 |
| CDC_Desire | - | + | U | + | + | 10.388 |
| AAC_Marchwell | - | + | + | + | + | 1.647 |
| AAC_Raymore | + | + | + | - | + | 6.605 |
| AAC_Spitfire | - | + | + | + | + | 3.986 |
| Transcend | - | + | + | + | + | 8.542 |
| CDC_Alloy | - | + | - | - | + | 2.439 |
| AAC_Durafield | - | + | - | + | + | 3.078 |
| AC_Morse | - | - | + | - | + | 5.139 |
| AC_Avonlea | - | - | + | - | + | 5.124 |
| Blackbird | + | + | + | + | + | 1.113 |
| Brigade | - | + | + | + | + | 1.740 |
| Commander | - | + | - | - | + | 6.733 |
| DT696 | - | + | + | + | + | 1.792 |
| Eurostar | - | - | + | - | + | 2.853 |
| R7_3_1 | + | - | - | - | + | 9.827 |
| Kronos | + | U | + | - | - | 4.261 |
| Langdon | - | - | - | + | + | 10.485 |
| Strongfield | - | + | + | + | + | 14.642 |
| CDC_Verona | - | - | + | - | + | 3.228 |
| AC_Navigator | - | + | - | U | + | 10.521 |
| A1200J_101 | + | + | + | + | + | 1.196 |
| A1200K_209 | + | + | + | + | + | 3.521 |
| DBC_144_12_14 | + | - | + | + | + | 4.054 |
| DBC_144_12_15 | + | - | + | + | + | 4.077 |
| DBC_144_17_1 | + | - | + | + | + | 12.951 |
| DBC_480_1 | + | - | + | + | + | 2.276 |
| DBC_480_10_2 | + | - | + | + | + | 1.166 |
| DBC_480_fam_3 | + | - | + | + | + | 2.326 |
| DN104_1324ABC | + | + | + | + | + | 11.547 |
| DN1089 | + | + | + | + | + | 22.695 |
| DT1011 | + | U | + | + | + | 1.602 |
| DT1016 | - | + | + | U | + | 1.691 |
| DT1019 | - | - | + | + | + | 1.761 |
| DT2004 | - | + | + | + | + | 7.439 |
| AAC_Grainland | - | + | - | + | + | 1.091 |
| DT881 | - | + | + | + | + | 4.215 |
| DT887 | - | + | + | + | + | 8.800 |
| DT890 | - | + | + | + | + | 6.687 |
| Durofo | + | - | - | - | + | 20.628 |
| Flora_111_5 | + | + | + | + | + | 3.309 |
| Flora_32_9 | + | + | + | + | + | 4.815 |
| Heli_52_1 | + | - | + | + | + | 0.548 |
| Heli_94_9 | + | - | + | - | + | 0.569 |
| I19_32_1 | + | - | + | + | + | 3.506 |
| I19_65 | + | - | + | + | + | 2.728 |
| Karur | + | - | + | U | + | 14.858 |
| P17_38 | + | - | + | + | + | 0.842 |
| P17_22 | + | - | - | + | + | 0.330 |
| P17_66 | + | - | - | - | + | 5.363 |
| P18_84 | + | - | + | + | + | 1.157 |
| R2_1_1 | + | - | + | + | + | 4.290 |
| R3_7_3 | + | - | + | + | + | 0.786 |
| R7_44_2 | + | - | + | - | + | 9.073 |
| R7_56_1 | + | - | - | - | + | 2.847 |
| R7_57_1 | + | - | - | - | + | 10.042 |
| Stelladur | + | - | + | + | + | 7.635 |
| DBC_263_30_1 | + | - | + | + | + | 4.253 |
| DN109_1382AC | + | + | + | + | + | 6.967 |
| DN123_1440ABC | - | + | + | + | + | 4.874 |
| DN136_1426AB | + | + | + | + | + | 8.775 |
| DN901_1363BD | + | + | + | + | + | 15.587 |
| DT1017 | + | - | + | + | + | 7.786 |
| DT1018 | + | - | + | U | + | 12.161 |
| DT2009 | - | + | + | + | + | 3.300 |
| DT2014 | - | + | U | + | + | 1.500 |
| DT896 | + | - | + | U | + | 2.073 |
| Flora_6_11 | + | + | + | + | + | 2.606 |
| Heli_31_1 | + | + | + | + | + | 3.493 |
| I19_9 | + | - | + | + | + | 1.207 |
| A1200H_005 | - | + | + | + | + | 1.625 |
| DN113_1390AB | + | + | - | + | + | 8.462 |
| DN125_1459ABC | + | + | + | + | + | 5.374 |
| DT1006 | + | + | - | U | + | 3.238 |
| DT1013 | + | U | + | - | + | 29.571 |
| DT1014 | - | + | + | + | + | 2.476 |
| DT1020 | - | + | U | + | + | 4.398 |
| DT1021 | + | + | U | + | + | 4.357 |
| DT879 | - | + | + | + | + | 3.925 |
| P33_30 | + | - | - | - | + | 17.111 |
| P33_34 | + | - | + | + | + | 5.842 |
| P33_51 | + | - | + | - | + | 2.018 |
| P49_57 | + | - | + | + | + | 1.200 |
| R11_11_1 | + | + | + | - | + | 10.273 |
| R7_60_1 | + | - | - | - | + | 8.447 |
| DN905_1434AB | + | + | + | + | + | 11.393 |
| DT1009 | + | + | + | - | - | 8.726 |
| DT1012 | U | + | + | U | - | 12.930 |
| DT1015 | + | - | + | + | - | 7.414 |
| Flora_122_2 | + | + | + | - | + | 1.667 |
| P17_54 | + | - | - | + | + | 1.519 |
| DT2017 | - | U | + | + | + | 6.500 |
| Heli_64_7 | + | - | + | - | + | 2.253 |
| I17_122_1 | + | - | + | + | + | 2.823 |
| I19_28_1 | + | - | + | + | + | 1.550 |
| P17_29 | + | - | - | + | + | 4.704 |
| P17_56 | + | - | - | - | + | 5.653 |
| P32_32 | + | - | + | + | + | 19.053 |
| Pescadou | - | - | - | + | + | 5.563 |
| R3_31_1 | + | - | + | + | + | 2.383 |
| DBC_144_12_16 | + | - | + | + | + | 23.813 |
| DT1010 | - | + | - | + | + | 19.881 |
| Heli_9 | + | - | + | + | + | 2.056 |
| SZD1029K | + | + | + | - | + | 8.121 |
| R13_18_1 | U | + | + | + | - | 15.541 |
| Levante | + | + | - | + | + | 11.927 |
| Capdur | + | + | - | + | + | 5.595 |
| DT735 | - | + | + | + | + | 1.610 |
| R2_44_2 | + | - | + | - | + | 6.588 |
| R3_22_1 | + | - | + | + | + | 0.674 |
| R14_18_1 | + | - | + | - | + | 2.452 |
| Durobonus | + | - | - | - | + | 32.147 |
| P17_10 | + | - | - | + | + | 3.120 |
| P17_15 | + | - | - | U | + | 4.270 |
| P17_43 | + | - | + | + | + | 2.339 |
| P17_44 | + | - | - | - | + | 14.864 |
| P17_51 | + | - | + | - | + | 11.624 |
| P17_52 | + | - | - | + | + | 2.052 |
| P17_58 | + | - | + | - | + | 16.748 |
| P17_63 | + | - | - | - | + | 33.128 |
| P17_7 | + | - | - | + | + | 4.356 |
| P17_8 | + | - | + | + | + | 10.239 |
| P18_10 | + | - | + | + | + | 1.993 |
| P18_13 | + | - | + | - | + | 2.131 |
| P18_15 | + | - | + | + | + | 12.883 |
| P18_23 | + | - | + | - | + | 6.000 |
| P18_26 | + | - | + | - | + | 2.729 |
| P18_33 | + | - | + | - | + | 7.813 |
| P18_4 | + | - | + | - | + | 1.159 |
| P18_71 | + | - | + | + | + | 16.770 |
| P18_80 | + | - | + | + | + | 1.600 |
| P17_20 | + | - | + | + | + | 1.658 |
| P17_32 | + | - | + | - | + | 22.821 |
| P17_47 | + | - | + | + | + | 2.140 |
| P17_48 | + | - | - | + | + | 7.583 |
| P17_9 | + | - | - | - | + | 7.549 |
| P18_12 | + | - | + | + | + | 2.152 |
| P18_55 | + | - | + | - | + | 17.950 |
| P18_62 | + | - | + | + | + | 5.895 |
| P18_7 | + | - | + | - | + | 11.055 |
| P18_76 | + | - | + | - | + | 25.548 |
| P32_14 | + | - | + | - | - | 3.159 |
| P32_3 | + | - | + | + | + | 7.016 |
| P17_36 | + | - | + | - | + | 2.979 |
| P18_38 | + | - | + | + | + | 12.476 |
| P18_73 | + | - | + | - | + | 2.362 |
| P32_30 | + | - | - | + | - | 2.800 |
| P32_37 | + | - | + | + | + | 23.895 |
| P32_38 | + | - | - | + | - | 6.412 |
| P32_4 | + | - | + | + | - | 10.216 |
| P32_45 | + | - | - | + | - | 11.284 |
| P32_53 | + | - | - | - | - | 20.881 |
| P32_55 | + | - | + | + | + | 10.537 |
| P32_66 | + | - | - | + | - | 9.576 |
| P32_69 | + | - | + | - | - | 1.961 |
| P32_7 | + | - | - | + | + | 6.357 |
| P33_10 | + | - | + | - | + | 3.914 |
| P33_12 | + | - | - | - | + | 10.959 |
| P33_19 | + | - | - | - | + | 23.927 |
| P33_2 | + | - | - | + | - | 34.381 |
| P33_23 | + | - | + | - | - | 38.780 |
| P33_25 | + | - | + | + | + | 5.110 |
| P33_27 | + | - | + | + | + | 23.488 |
| P33_31 | + | - | - | + | - | 6.050 |
| P33_33 | + | - | + | + | + | 2.439 |
| P33_50 | + | - | - | U | - | 19.996 |
| P33_36 | + | - | - | - | + | 30.631 |
| P33_39 | + | - | - | - | + | 31.820 |
| P33_41 | + | - | + | - | - | 17.207 |
| P33_44 | + | - | + | - | - | 8.289 |
| P33_53 | + | - | + | - | + | 16.384 |
| P33_6 | + | - | + | - | - | 6.853 |
| P33_63 | + | - | + | + | - | 2.133 |
| P33_8 | + | - | - | + | - | 7.437 |
| P48_10 | + | - | + | + | + | 4.757 |
| P48_13 | + | - | + | - | + | 5.007 |
| P48_23 | + | - | + | - | + | 2.887 |
| P48_32 | + | - | + | + | + | 5.171 |
| P48_35 | + | - | + | - | + | 12.851 |
| P48_41 | + | + | + | - | + | 3.458 |
| P48_5 | + | + | + | + | + | 6.459 |
| P48_9 | + | - | + | + | + | 5.448 |
| P49_12 | + | + | + | + | + | 1.539 |
| P49_15 | + | - | + | - | + | 17.699 |
| P49_30 | + | - | + | - | + | 11.645 |
| P49_37 | + | + | + | - | + | 11.034 |
| P49_39 | + | - | + | + | + | 3.485 |
| P49_4 | + | - | + | - | + | 4.206 |
| P49_43 | + | + | + | + | + | 2.470 |
| P49_45 | + | - | + | + | + | 4.428 |
| P49_48 | + | - | + | - | + | 8.059 |
| P49_51 | + | - | - | + | + | 2.008 |
| P49_60 | + | + | + | - | + | 28.935 |
| P49_68 | + | - | + | - | + | 1.998 |
| P49_7 | + | + | + | + | + | 5.342 |
| R11_27_1 | + | + | + | + | + | 1.113 |
| R11_34_1 | + | U | + | + | + | 9.507 |
| R11_48_2 | + | - | + | - | + | 8.398 |
| R12_11_1 | + | + | - | + | + | 5.159 |
| R13_4_2 | + | - | - | + | + | 14.848 |
| R14_21_1 | + | - | + | + | + | 2.074 |
| R2_42_3 | + | - | + | - | + | 5.121 |
| R3_12_1 | + | - | - | + | + | 2.943 |
| R7_54_2 | + | - | + | - | + | 2.851 |
| R7_61_1 | + | + | U | - | + | 1.719 |
| R7_63_2 | + | - | + | + | + | 3.949 |
| P32_41 | + | - | - | + | + | 32.033 |
| P32_51 | + | - | - | + | - | 1.852 |
| P32_54 | + | - | - | + | - | 6.175 |
| P32_62 | + | - | - | + | - | 4.749 |
| P33_21 | + | - | + | - | - | 8.961 |
| P33_42 | + | - | U | - | + | 6.981 |
| P33_48 | + | - | - | - | - | 4.428 |
| P33_64 | + | - | - | + | + | 10.095 |
| P48_4 | + | - | + | - | + | 4.294 |
| R11_17_1 | + | - | + | + | + | 4.553 |
| R11_26_1 | + | - | + | + | + | 2.795 |
| R2_41_1 | + | - | + | - | + | 6.923 |
| R3_11_3 | + | - | + | - | + | 1.562 |
| R7_1_1 | + | - | + | - | + | 3.212 |
| R7_11_2 | + | U | + | - | + | 3.666 |
| R7_14_1 | + | - | + | - | - | 2.197 |
| R7_19_2 | + | - | + | - | + | 9.393 |
| R7_20_1 | + | - | + | - | + | 14.119 |
| R7_25_1 | + | + | - | - | - | 1.493 |
| R7_28_3 | + | + | + | - | + | 1.884 |
| R7_40_1 | + | - | + | - | + | 3.262 |
| R7_50_1 | + | - | + | - | + | 2.960 |
| R7_51_1 | + | - | + | - | + | 17.874 |
| R7_6_2 | + | - | - | - | + | 5.360 |
| P49_1 | + | - | + | + | + | 3.625 |
| P49_64 | + | - | + | + | + | 5.229 |
| R11_29_1 | U | + | + | + | + | 2.271 |
| R2_19_3 | + | - | + | U | + | 3.433 |
| R2_32_1 | + | + | + | U | + | 25.489 |
| R7_21_3 | + | - | + | + | + | 10.586 |
| R7_33_1 | + | - | U | + | + | 1.867 |
| R7_35_2 | + | - | + | - | + | 3.733 |
| R7_41_1 | U | + | - | - | + | 25.732 |
| P18_40 | + | - | + | - | + | 8.239 |
| P48_19 | + | + | + | + | + | 6.256 |

“+”, resistance allele of the corresponding QTL; “-”, susceptibility allele of the corresponding QTL; “U”, uncertain
